# G-CSF resistance of *ELANE*-mutant neutropenia depends on SERF1-containing truncated–neutrophil elastase aggregates

**DOI:** 10.1172/JCI177342

**Published:** 2024-11-19

**Authors:** Ramesh C. Nayak, Sana Emberesh, Lisa R. Trump, Ashley M. Wellendorf, Abhishek K. Singh, Brice Korkmaz, Marshall S. Horwitz, Kasiani C. Myers, Theodosia A. Kalfa, Carolyn M. Lutzko, Jose A. Cancelas

**Affiliations:** 1 Hoxworth Blood Center, University of Cincinnati College of Medicine, Cincinnati, Ohio, USA.; 2 Division of Experimental Hematology and Cancer Biology, Cancer & Blood Diseases Institute, Cincinnati Children’s Hospital Medical Center, University of Cincinnati College of Medicine, Cincinnati, Ohio, USA.; 3 INSERM UMR-1100, Research Center for Respiratory Diseases, and University of Tours, Tours, France.; 4 Department of Laboratory Medicine & Pathology, University of Washington School of Medicine, Seattle, Washington, USA.; 5 Connell and O’Reilly Families Cell Manipulation Core Facility & Department of Medical Oncology, Dana-Farber Cancer Institute, Harvard Medical School, Boston, Massachusetts, USA.

**Keywords:** Hematology, Neutrophils

## Abstract

Severe congenital neutropenia (SCN) is frequently associated with dominant point mutations in *ELANE*, the gene encoding neutrophil elastase (NE). Chronic administration of granulocyte colony–stimulating factor (G-CSF) is a first-line treatment of *ELANE*-mutant (*ELANE*^mut^) SCN. However, some *ELANE*^mut^ patients, including patients with *ELANE* start codon mutations, do not respond to G-CSF. Here, through directed granulopoiesis of gene-edited isogenic normal and patient-derived iPSCs, we demonstrate that *ELANE* start codon mutations suffice to induce G-CSF–resistant granulocytic precursor cell death and refractory SCN. *ELANE* start codon–mutated neutrophil precursors express predominantly nuclear N-terminally truncated alternate NE. Unlike G-CSF–sensitive *ELANE* mutations that induce endoplasmic reticulum and unfolded protein response stress, we found that the mutation of the *ELANE* translation initiation codon resulted in NE aggregates and activated proapoptotic aggrephagy, as determined by downregulated BAG1 expression, decreased BAG1/BAG3 ratio, NE colocalization with BAG3, and localized expression of autophagic LC3B. We found that SERF1, an RNA-chaperone protein, known to localize in misfolded protein aggregates in neurodegenerative diseases, was highly upregulated and interacted with cytoplasmic NE of mutant neutrophil precursors. Silencing of SERF1 enhanced survival and differentiation of iPSC-derived neutrophil precursors, restoring their responsiveness to G-CSF. These observations provide a mechanistic insight into G-CSF–resistant *ELANE*^mut^ SCN, revealing targets for therapeutic intervention.

## Abstract 2

Start codon mutations of ELANE result in aggregates of truncated NE that initiate aggrephagy and resistance to G-CSF, reversible by silencing SERF1.

## Graphical abstract

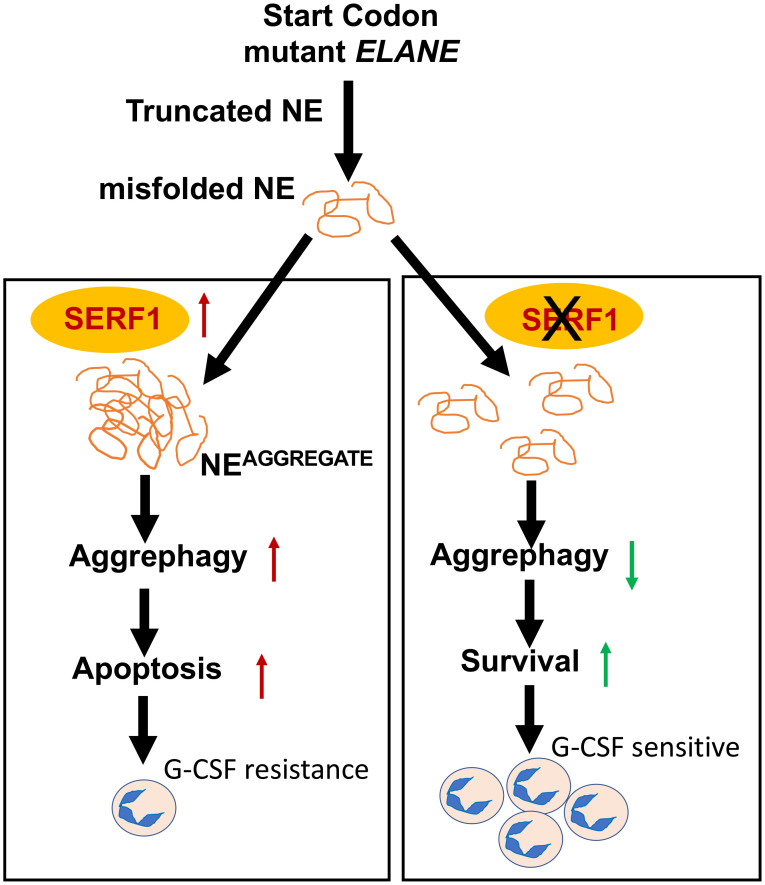

## Introduction

Germline heterozygous point mutations in the gene *ELANE*, which encodes neutrophil elastase (NE), are the most frequent cause of severe congenital neutropenia (SCN). SCN results from cell death and maturation arrest of bone marrow neutrophil precursors (1–4). Although granulocyte colony–stimulating factor (G-CSF) administration increases peripheral blood (PB) absolute neutrophil counts (ANCs) in most *ELANE*^mut^ SCN patients (5), patients with mutations involving the translation initiation codon of the *ELANE* gene are resistant to G-CSF administration (6–8). Moreover, up to a quarter of *ELANE*^mut^ SCN cases on long-term, high-dose G-CSF therapy develop somatic colony-stimulating factor 3 receptor–activating (CSF3R-activating) mutations preceding the onset of myelodysplastic syndromes (MDS) and acute myeloid leukemia (AML) (9), underscoring the need to investigate alternative combinatorial therapies that could decrease therapeutic reliance on G-CSF.

The pathogenesis of many cases of *ELANE*^mut^ SCN pivots around mislocalization, impaired endoplasmic reticulum (ER) trafficking of mutant NE, and induction of unfolded protein response (UPR) pathways that lead to death and differentiation arrest of neutrophilic precursors (2, 10, 11). Previously, we showed that mutation at the translation initiation codon of *ELANE* results in alternate NE (N-terminally truncated) expression translating from downstream in-frame start codons (12). However, the molecular and cellular mechanisms of the pathogenesis and G-CSF resistance of *ELANE* translation initiation codon–mutant SCN has been largely unknown.

In this study, we modeled G-CSF–resistant SCN associated with *ELANE* translation initiation codon mutations using patient-derived and gene-edited induced pluripotent stem cells (iPSCs). We found that the mutation at the *ELANE* translation initiation codon results in expression and aggregation of N-terminally truncated alternate NE peptides, induction of exaggerated aggrephagy, along with upregulation of SERF1. We showed that downregulation of SERF1 prevents aggregate formation and rescues granulocytic precursor survival and differentiation. Our study illustrates an alternate mechanism of protein aggregation–mediated pathobiology of *ELANE* translation initiation codon–mutant SCN.

## Results

### ELANE translation initiation codon mutation induces apoptosis and impairs differentiation.

**Figure 1:**
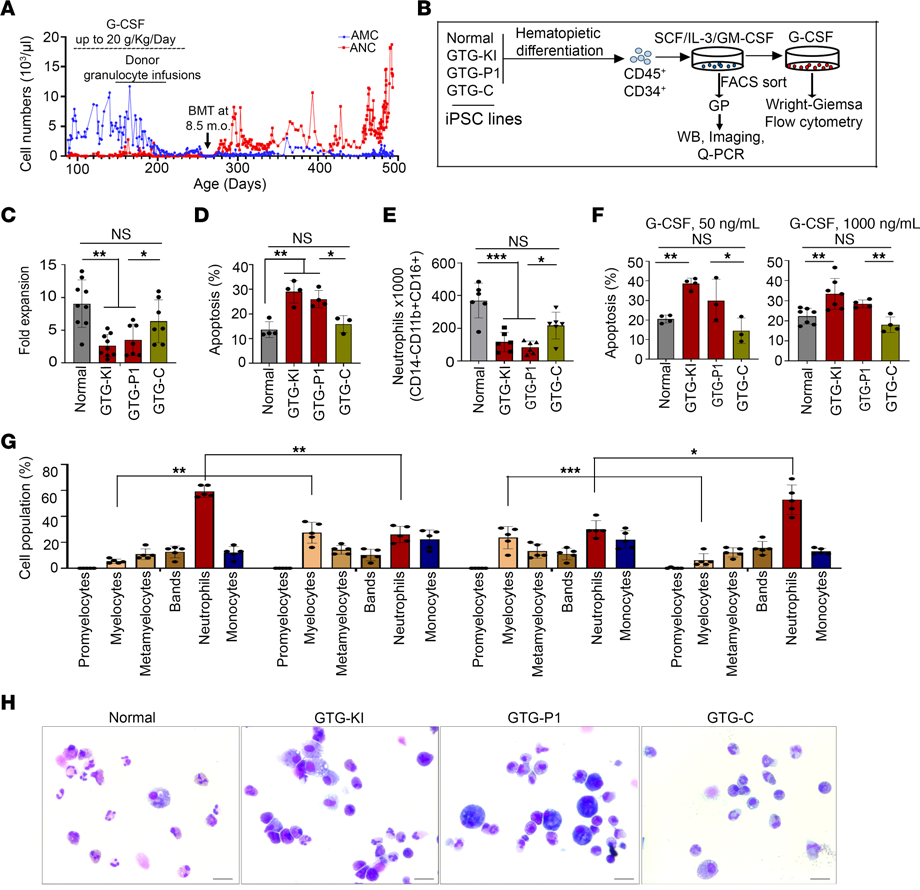
Neutrophil precursors with mutation at the translation initiation codon (c.1A>G) of *ELANE* that is associated with cell death, differentiation arrest, and G-CSF resistance. (**A**) PB ANCs and AMCs of a patient with *ELANE* translation initiation codon mutation (c.1A>G, NEp.M1V) from diagnosis at 3 months after birth up to 16 months. The patient was found to have leukopenia, severe neutropenia (ANC = 0), and monocytosis (AMC = 2720/mm^3^). G-CSF (20 μg/kg/day) administration showed minimal effect on ANCs. Donor granulocyte transfusion did not increase neutrophil counts. This patient underwent allogeneic bone marrow transplantation, resulting in ANC recovery. (**B**) Experimental schema of modeling of SCN with *ELANE* translation initiation codon mutation employing directed hematopoietic and granulopoietic differentiation of normal (healthy donor iPSCs*),* isogenic gene-edited GTG-KI (GTG knockin in place of ATG in one allele of *ELANE* gene of normal iPSCs), GTG-P1 (*ELANE* c.1A>G SCN patient–derived iPSCs), and GTG-C (correction of GTG to ATG in SCN patient GTG-P1 iPSCs). (**C**) Cell growth of normal, GTG-KI, GTG-P1, and GTG-C iPSC–derived hematopoietic progenitors (CD34^+^CD45^+^). (**D**) Quantification of the apoptosis of normal, GTG-KI, GTG-P1, and GTG-C neutrophil precursors (CD45^+^CD34^–^CD14^–^CD11b^–^CD15^+/dim^). (**E**) Flow cytometry analyses of the granulopoiesis (mature neutrophils: CD45^+^CD14^–^CD11b^+^CD16^+^CD66b^+^) of normal, GTG-KI, GTG-P1, and GTG-C iPSC–derived hematopoietic progenitors in the presence of 50 ng/mL G-CSF. (**F**) Quantification of the apoptosis of normal, GTG-KI, GTG-P1, and GTG-C neutrophil precursors (CD45^+^CD34^–^CD14^–^CD11b^–^CD15^+^) in the presence of 50 ng/mL and 1000 ng/mL G-CSF. (**G** and **H**) Quantification (**G**) and representative morphological microphotographs (**H**) of output cells of G-CSF–induced (50 ng/mL) differentiation of normal, GTG-KI, GTG-P1, and GTG-C iPSC–derived hematopoietic progenitors (Wright-Giemsa staining; original magnification, ×40). Data are presented as individual data and mean ± standard deviation of a minimum of 3 replicates per experiment and a minimum of 2 independent experiments. Differences between groups were evaluated using 1-way ANOVA. *P < 0.05; **P < 0.01; ***P < 0.001.

In the present study, we modeled and investigated the mechanisms underlying G-CSF–resistant SCN pathobiology with mutations at the translation initiation codon of *ELANE*. We reprogrammed iPSCs from PB mononuclear cells (MNCs) of healthy individuals (normal) and 2 SCN patients (GTG-P1 and ATA-P2) with heterozygosity for *ELANE* c.1A>G, p.(M1V) and *ELANE* c.3G>A, p.(M1I), respectively. As illustrated in Figure 1A, SCN patient 1, who donated the source cells to generate GTG-P1 iPSCs, did not respond to G-CSF therapy (20 mg/kg/day) and his neutrophil count only recovered after allogeneic hematopoietic stem cell transplantation (HSCT). Two half-siblings of this patient, who were also diagnosed with SCN due to the same M1V mutation, did not respond to G-CSF therapy either (at a dose of 10 and up to 75 mg/kg/day), and also required HSCT (7, 8). ATA-P2 was a female diagnosed with SCN in the first year of life. She had been treated off and on with recombinant G-CSF throughout her life, with poor response and no evidence of cycling. While receiving maximal tolerable dosing of G-CSF on a daily weekday regimen (Monday through Friday), her total white blood cell count reached up to approximately 2,600 per mm^3^, with an ANC of 234 per mm^3^ and absolute monocyte count (AMC) of 832 per mm^3^.

To study whether the *ELANE* translation initiation codon mutation is sufficient and necessary to induce SCN pathogenesis, we additionally generated isogenic knockin (GTG-KI) cells, where we introduced the mutation c.1A>G in healthy donor (normal) iPSCs and we corrected the mutation in GTG-P1 (SCN patient–derived iPSC line), named as the GTG-C iPSC line (Supplemental Figure 1A; supplemental material available online with this article; https://doi.org/10.1172/JCI177342DS1). The patient-derived and knockin iPSC lines retained the mutation c.1A>G, while CRIPSR/Cas9-mediated homology-directed repair (HDR) led to correction of the heterozygote mutation in the GTG-C iPSC line (Supplemental Figure 1B). All iPSC lines maintained pluripotency and normal karyotype throughout the culture period (Supplemental Figure 1, C and D).

iPSC lines were subjected to directed hematopoietic differentiation and generated similar levels of hematopoietic progenitors (CD45^+^CD34^+^) between isogenic iPSC lines, and the majority of these cells express CD43, a marker for primitive hematopoiesis (Supplemental Figure 2, A and B). Hematopoietic progenitors from iPSC lines were cultured in ex vivo granulopoiesis conditions (10) (Figure 1B). The growth of CD34-negative neutrophil precursors in ex vivo granulopoiesis starting from mutation-knockin (GTG-KI) and patient-generated (GTG-P1) iPSCs were reduced by approximately 75% and approximately 50%, respectively, compared with normal control (Figure 1C). The reduced cell growth was associated with increased apoptosis (Figure 1D and Supplemental Figure 3, A and B), similar to other models of mutations in *ELANE* (10, 13). Gene correction of GTG-P1 iPSCs (GTG-C) restored cell growth and survival to levels comparable to normal iPSC–derived precursors (Figure 1C and Supplemental Figure 3, A and B). To examine whether SCN patient iPSC–derived granulocytic precursors recapitulate the disease observed in SCN patients, we performed assays of in vitro–directed granulopoiesis in the presence of G-CSF at low (50 ng/mL) and high (1000 ng/mL) concentrations, as described previously (10), which roughly corresponds to peak plasma concentrations of G-CSF in infants receiving 1 or 20 μg/kg/day, respectively (14). During G-CSF–directed granulopoiesis, cultures of *ELANE* c.1A>G neutrophil precursors contained fewer cells (Figure 1E) and had increased apoptosis both at low and high concentrations of G-CSF (Figure 1F). The granulocytic differential count of mutation knockin (GTG-KI) and patient (GTG-P1) iPSC–derived myeloid precursors was shifted left, with significantly reduced levels of mature neutrophils (CD45^+^CD14^–^CD11b^+^CD15^+^CD66b^+^) in both low and high levels of G-CSF, as assessed by flow cytometry (Supplemental Figure 3, C–E) and by morphological analysis (Figure 1, G and H). High concentration of G-CSF did not ameliorate the defects in granulopoietic differentiation in the GTG-KI and GTG-P1 lines (Supplemental Figure 3, F and G). These data suggest that *ELANE*^mut^ SCN iPSC–derived myeloid precursor cells are arrested at the myelocyte stage of granulopoietic development even at suprapharmacological concentrations of G-CSF. Survival and granulocytic maturation were restored to normal levels in mutation-corrected (GTG-C) neutrophil precursors (Figure 1, D–H, and Supplemental Figure 3, C–G) without affecting the surface expression of CSF3R (Supplemental Figure 3H). Analysis of 2 other clones of healthy donor, mutant, knockin, and corrected iPSC–derived hematopoietic progenitors (CD45^+^CD34^+^CD43^+^) (Supplemental Figure 4, A and B, and Supplemental Figure 5, A–C) further supported these findings and the effect of the translation initiation codon mutation on granulopoiesis failure both at low and high concentration of G-CSF (Supplemental Figure 5, A–C, and Supplemental Figure 6, A–D). These results, taken together, demonstrate that *ELANE* translation initiation codon mutation is necessary and sufficient to recapitulate human SCN pathogenesis characterized by maturation arrest, cell death, and G-CSF resistance.

### Neutrophil precursors with ELANE c.1A>G mutation express N-terminally truncated NE protein, which aggregates and induces apoptosis preferentially via aggrephagy rather than UPR/ER stress like most other ELANE mutations.

**Figure 2:**
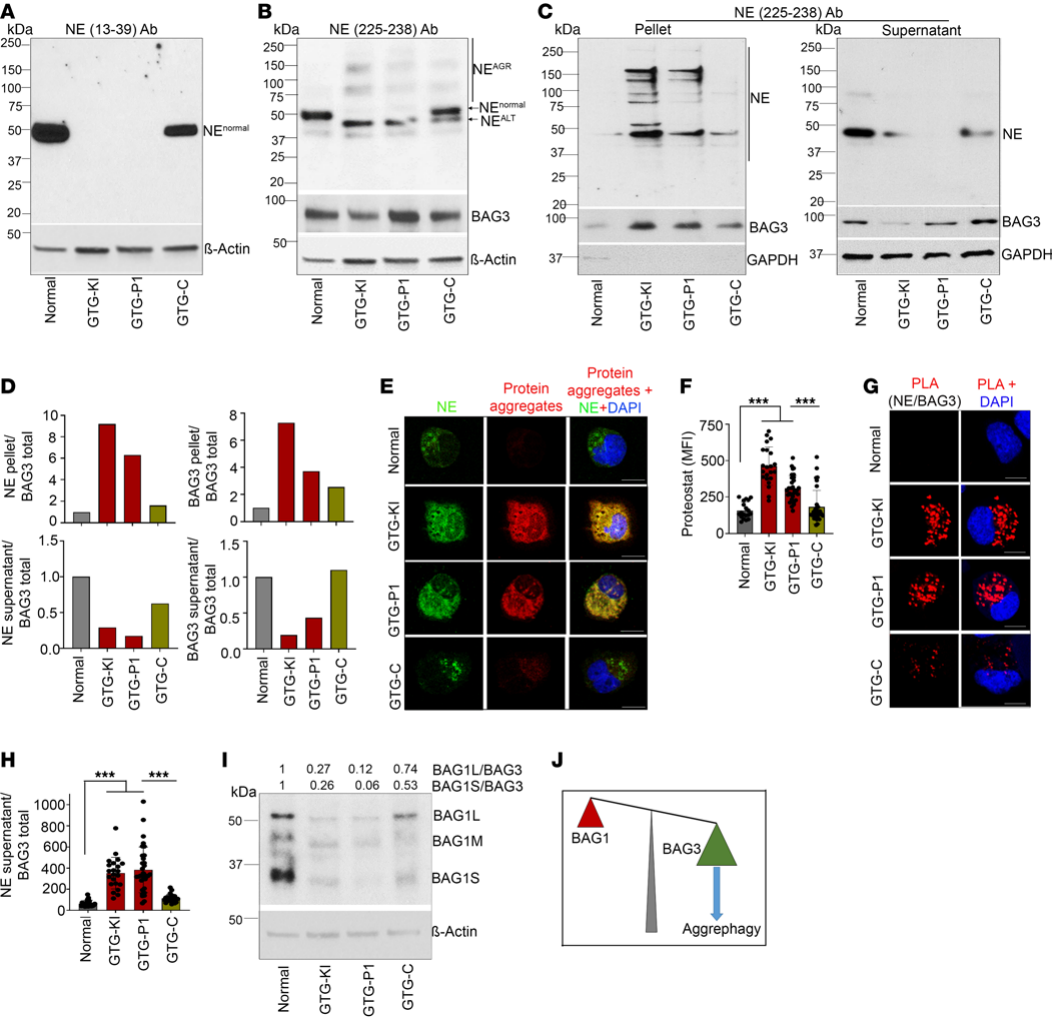
Neutrophil precursors with mutation at translation initiation codon of *ELANE* express low molecular weight alternate NE (NE^ALT^), generate NE aggregates (NE^AGR^), and induce aggrephagy. (**A**) Representative immunoblot analyses of full-length NE (NE^Normal^) expression using α-NE Ab against N-terminal region (aa 13–39) of NE. (**B**) Representative immunoblot of NE expression in neutrophil precursors derived from normal, GTG-KI, GTG-P1, and GTG-C iPSC lines using an α-NE Ab against the C-terminal (aa 225–238) region of the protein. GTG-KI and GTG-P1 iPSC–derived neutrophil precursors express alternate NE (NE^ALT^) and possibly aggregates (NE^AGR^) of alternate NE peptides, and mutation correction rescues full-length NE expression. Some of the NE^ALT^ and NE^AGR^ expression in the GTG-C line could be due to contamination of mutant iPSCs during clonal isolation. (**C**) Representative immunoblots f NE in the pellet and soluble fractions of granulocyte precursor lysates after ultracentrifugation. (**D**) Quantification of NE and BAG3 in **C** in pellets and soluble fractions and presented as a normalized ratio over BAG3 (from panel **B**). (**E**) Representative confocal microscopic images of NE in association with ProteoStat fluorescent molecular rotor dye. (**F**) MFI of ProteoStat-stained aggresomes. (**G**) Representative immunoblots of BAG1 isoforms (BAG1L, BAG1M, and BAG1S) in normal, GTG-KI, GTG-P1, and GTG-C neutrophil precursors. (**H** and **I**) Representative confocal microscopic images (**H**) and MFI (**I**) of proximity ligation assay (PLA) signal between NE and BAG3 in normal, GTG-KI, GTG-P1, and GTG-C neutrophil precursors. (**J**) Schematic depiction of BAG3 and BAG1 protein ratio and association with NE^ALT^ aggregation. Scale bars: 10 μm. The MFIs of more than 10 cells from 2 independent experiments were quantified. Data are presented as individual data and mean ± standard deviation of 2 or 3 replicates per experiment and a minimum of 2 independent experiments. Differences between groups were evaluated using 1-way ANOVA. ***P < 0.001.

To determine whether start-codon-mutant ELANE resulted in a loss of the N-terminus of NE, we compared immunoblots using an antibody specific against the C-terminal region of NE (Supplemental Figure 7, A and B) and a specific antibody directed against the N-terminus of NE (Figure 2A). Consistent with our earlier report (12), neutrophil precursors (CD45^+^CD34^–^CD11b^–^CD14^–^CD15^+^) derived from *ELANE* translation initiation codon–mutant (GTG-KI and GTG-P1) iPSCs expressed N-terminally truncated low molecular weight alternate NE (NE^ALT^) and higher molecular weight NE bands possibly due to misfolding and aggregation (NE^AGR^) of the truncated alternate NE (Figure 2, A and B). As expected, cellular NE distribution was altered in mutant neutrophil precursors (12). While NE was predominantly cytoplasmic in healthy donor and genetically corrected neutrophil precursors, most neutrophil precursors (~60%) displayed a predominantly nuclear distribution of NE (Supplemental Figure 7, C and D). Healthy donor (normal) and mutation-corrected (GTG-C) neutrophil precursors predominantly expressed full-length NE (NE^Normal^) (Figure 2, A and B, and Supplemental Figure 8, A and B). A similar pattern of low molecular weight and aggregated forms of NE was observed in neutrophil precursors from the ATA-P2 iPSC line carrying the translation initiation codon c.3G>A mutation (Supplemental Figure 8C). Non-mutant NE or *ELANE* exon 3 (*ELANE*^EX-3^) missense mutation (NE^I118N^ and NE^Q96P^) SCN patient iPSC–derived neutrophil precursors did not express NE^ALT^ and NE^AGR^ (Supplemental Figure 8D), suggesting a phenomenon specific to translation initiation codon mutations. Of note, no significant changes in the expression of Bcl2-associated athanogene-3 (BAG3), a co-chaperone critical for the aggrephagy of misfolded proteins (15), were associated with the presence of the *ELANE* translation initiation codon mutation (Figure 2B).

To determine whether the high molecular weight bands detected by immunoblotting with anti-NE C-terminal region–specific antibody are indeed aggregated forms of mutant NE, we performed cell fractionation using ultracentrifugation (200,000*g*) to separate insoluble and aggregated proteins from supernatant containing soluble proteins. Fractionation of GAPDH exclusively in the supernatant fraction suggested that native proteins remain in the soluble fraction at 200,000*g* (Figure 2C). As shown in Figure 2, C and D, NE and BAG3 from GTG-KI and GTG-P1 iPSC–derived cells were highly insoluble and predominantly present in the pellet fractions. While pellet anti-BAG3 recognized a single band, pellet anti-NE recognized multiple high molecular weight bands. As expected, most NE from normal and mutation-corrected granulocytic precursors was enriched in the supernatant fraction (Figure 2, C and D). BAG3 was enriched in the pellet fractions of mutation-carrying cells, but not in healthy or corrected cells (Figure 2, C and D), further supporting the notion of induced aggrephagy in GTG-KI and GTG-P1 granulocyte precursors. The presence of NE-containing aggregates in *ELANE* translation initiation codon–mutant, but not in normal or mutation-corrected, neutrophil precursors was further confirmed by microscopy that demonstrated cytosolic colocalization of NE with the fluorescent dye ProteoStat (Figure 2, E and F, and Supplemental Figure 9, A and B), a fluorescent molecular rotor that detects misfolded and aggregated proteins (16, 17).

Unlike *ELANE*^EX-3^ mutations (10), neutrophil precursor cell death induced by the *ELANE* c.1A>G mutation did associate with increased expression of proapoptotic BH3-only molecules (Supplemental Figure 10A), but did not associate consistently with increased UPR/ER stress response (Supplemental Figure 10B). Intrigued by the lack of noticeable UPR/ER stress response, which was in clear contradiction with the behavior of other *ELANE* mutations (2, 10, 11), we hypothesized that an alternative cell death mechanism was at play. The aggregation of alternate NE peptides (Figure 2, A–F, Supplemental Figure 8C, and Supplemental Figure 9, A and B) was associated with enriched distribution of BAG3 into the pellet fraction and increased expression of autophagic LC3B (Supplemental Figure 10, C and D). Together, these data were strongly suggestive of the existence of aggrephagy activation in *ELANE* translation initiation codon–mutant (GTG-KI, GTG-P1, and ATA-P2) neutrophil precursors. To further validate the induction of aggrephagy in mutant precursors, we analyzed the level of expression of the BAG family proteins that act as co-chaperones of HSP70 family proteins (18, 19). The existence of aggrephagy activation in GTG-KI and GTG-P1 mutant myeloid precursors was further supported by decreased BAG1L/BAG3 and BAG1S/BAG3 ratios, a hallmark of aggrephagy pathway activation, and BAG3 interaction with NE^AGR^ in mutant cells, as demonstrated by proximity ligation assay (PLA) (Figure 2, G–J). We also found that the expression of all 3 isoforms of BAG1 (BAG1L, BAG1M, and BAG1S) was drastically reduced in GTG-KI and GTG-P1 neutrophil precursors (Figure 2I). Gene editing–mediated correction of the translation initiation codon mutation restored the expression of BAG1L, and to a lesser degree BAG1S, to normal or close-to-normal precursor expression levels (Figure 2I). BAG1 and BAG3 act as co-chaperones (18, 19) driving, respectively, the misfolded protein response toward proteasome-mediated degradation (20) or the macroautophagic process of aggrephagy (15, 21). Therefore, the BAG1 to BAG3 protein ratio dictates a proteasome to autophagy switch in neurodegenerative diseases (22–24) and has been shown to be critical for HSC homeostasis and aging (25). The reduced expression of BAG1 isoforms, decreased BAG1/BAG3 ratio, and association of BAG3 with NE supports our hypothesis of alternate NE peptide–induced aggrephagy machinery in these cells, a mechanism similar to neurodegenerative diseases (24).

### SERF1 is required for aggrephagy-induced cell death and G-CSF resistance of ELANE translation initiation–mutant neutrophil precursors.

**Figure 3:**
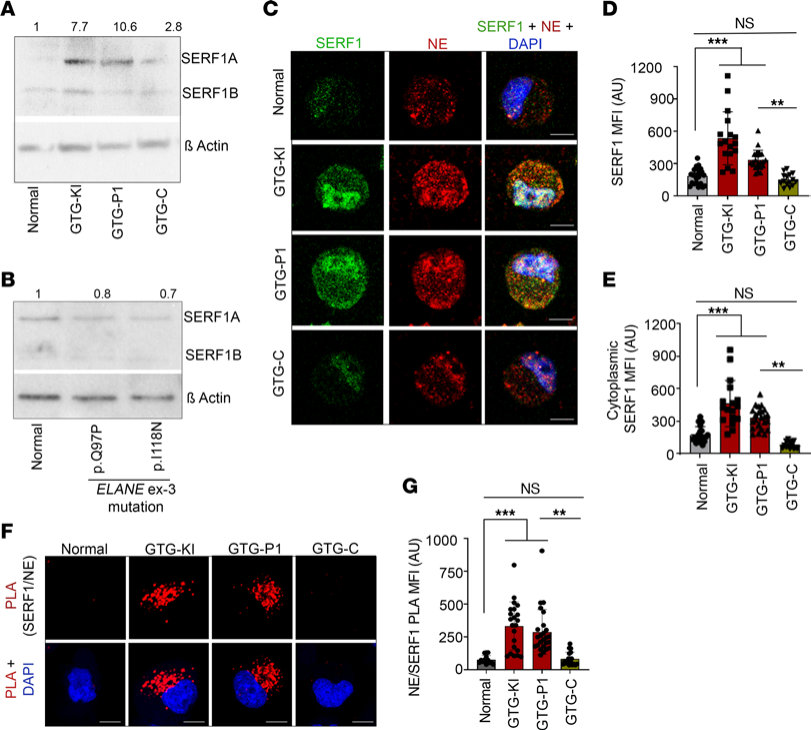
Expression of SERF1, an RNA binding chaperone, is upregulated and interacts with NE aggregates in *ELANE* translation initiation codon–mutant neutrophil precursors. (**A**) Representative immunoblots of SERF1A and SERF1B in normal, GTG-KI, GTG-P1, and GTG-C neutrophil precursors. SERF1A, but not SERF1B, expression is upregulated in *ELANE* translation initiation–mutant neutrophil precursors. (**B**) Representative Western blots of SERF1A and SERF1B in healthy donor (normal) and *ELANE*^EX-3^ mutant (I118N, Q97P) iPSC–derived neutrophil precursors. SERF1A expression is marginally reduced in *ELANE*^EX-3^ mutant SCN. (**C** and **D**) Representative confocal microscopic images of NE and SERF1 (**C**) and MFIs (**D**) of SERF1A in normal, GTG-KI, GTG-P1, and GTG-C neutrophil precursors. (**E**) Quantification of SERF1 cytoplasmic expression in normal, GTG-KI, GTG-P1, and GTG-C neutrophil precursors. Increased SERF1 expression is associated with translocation to cytoplasm. (**F** and **G**) Representative confocal images (**F**) and MFIs (**G**) of PLA signal between SERF1 and NE normal, GTG-KI, GTG-P1, and GTG-C neutrophil precursors. Scale bars: 10 μm. The MFIs of more than 10 cells from 2 independent experiments were quantified. Data are presented as individual data and mean ± standard deviation of 2 or 3 replicates per experiment and a minimum of 2 independent experiments. Differences between groups were evaluated using 1-way ANOVA. **P < 0.01; ***P < 0.001.

To understand the underlying mechanisms of aggrephagy-induced cell death and granulopoiesis arrest, we first examined the expression of known cellular chaperones. We found no change in the expression of heat shock proteins HSP90, HSP70, and HSP40, chaperones that regulate protein refolding to native states (Supplemental Figure 11A). The expression of SERF1A protein, known to regulate aggregation and proteotoxicity in neurodegenerative diseases (26–28), was significantly upregulated in *ELANE* c.1A>G, but not in *ELANE*^EX-3^ mutant, neutrophil precursors (Figure 3, A and B). SERF1 is an evolutionarily conserved human homolog of *C*. *elegans* MOAG4 protein (26) and regulates protein aggregation and age-related proteotoxicity (26–28). SERF1 is a nuclear stabilizer with capacity to bind RNA that translocates to the cytoplasm in response to stress generated by cytotoxic aggregates of misfolded proteins and binds to insoluble protein aggregates (29). As expected, we found SERF1 expression to be predominantly nuclear in normal and mutation-corrected iPSC–derived neutrophil precursors (Figure 3C). The increased SERF1 expression in *ELANE* translation initiation codon–mutant (GTG-KI and GTG-P1) neutrophil precursors was associated with both nuclear and cytoplasmic distribution (Figure 3, C–E). Consistent with our earlier report (12), we found NE located in both the cytoplasm and nucleus of granulocyte precursors (Figure 3C). PLA analysis between SERF1 and NE demonstrated that both proteins located in proximity in the cytoplasm of GTG-KI and GTG-P1 neutrophil precursors, but not in healthy control or mutation-corrected cells (Figure 3, F and G). Although we observed NE localization to the nucleus, we found no proximity between NE and SERF1 in the nucleus, suggesting that SERF1 did not interact with NE in the nucleus (Figure 3, F and G). As a control for specificity, the endogenous protease inhibitor α1-antitrypsin (AAT) was found to interact with NE in all the groups tested (Supplemental Figure 11B), but not with SERF1 (Supplemental Figure 11C), reinforcing the notion that the interaction between SERF1 and NE is specific and distinct from other known interactions in neutrophil precursors (30).

**Figure 4:**
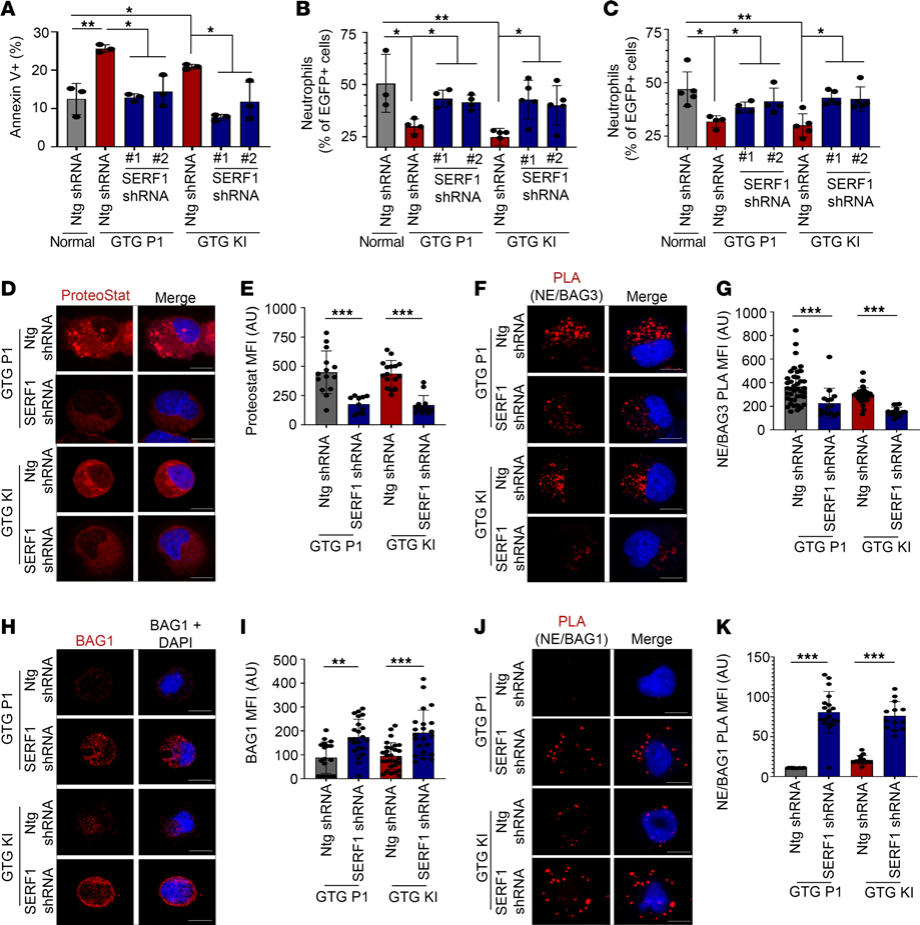
SERF1 downregulation restores survival and granulocytic differentiation of *ELANE* translation initiation codon–mutant neutrophil precursors. (**A**) Apoptosis of non-targeting (Ntg) shRNA– and SERF1 shRNA–transduced GTG-KI and GTG-P1 neutrophil precursors. SERF1 downregulation led to increased survival of GTG-KI and GTG-P1 neutrophil precursors. (**B**) Granulopoietic differentiation of Ntg shRNA– and SERF1 shRNA–transduced GTG-KI and GTG-P1 iPSC–derived hematopoietic progenitors at 50 ng/mL G-CSF. (**C**) Granulopoietic differentiation of Ntg shRNA– and SERF1 shRNA–transduced GTG-KI and GTG-P1 iPSC–derived hematopoietic progenitors at 1000 ng/mL G-CSF. (**D** and **E**) Representative confocal microscopic images (**D**) and MFIs (**E**) of ProteoStat fluorescent molecular rotor dye in Ntg shRNA– and SERF1 shRNA–transduced GTG-KI and GTG-P1 neutrophil precursors. (**F** and **G**) Representative confocal microscopic images (**F**) and MFIs (**G**) of PLA signals between NE and BAG3 in Ntg shRNA– and SERF1 shRNA–transduced GTG-KI and GTG-P1 neutrophil precursors. (**H** and **I**) Representative confocal microscopic images and MFIs of BAG1 expression in Ntg shRNA– and SERF1 shRNA–transduced GTG-KI and GTG-P1 neutrophil precursors. (**J** and **K**) Representative confocal microscopic images and MFIs of PLA between NE and BAG1. SERF1 downregulation enhanced NE interaction with BAG1 in GTG-P1 and GTG-KI neutrophil precursors. Scale bars: 10 μm. The MFIs of more than 10 cells from 2 independent experiments were quantified. Data are presented as individual data and mean ± standard deviation of 2 or 3 replicates per experiment and a minimum of 2 independent experiments. Differences between groups were evaluated using 1-way ANOVA. *P < 0.05; **P < 0.01; ***P < 0.001.

To determine whether SERF1 upregulation is essential for NE aggregation and SCN pathogenesis, we downregulated SERF1 expression using short hairpin RNA (Supplemental Figure 12, A–D). Downregulation of SERF1A rescued the survival (Figure 4A and Supplemental Figure 12E), differentiation, and G-CSF sensitivity of GTG-KI and GTG-P1 neutrophil precursors (Figure 4, B and C), suggesting that downregulation of SERF1 made the *ELANE* translation initiation codon–mutant neutrophil precursors sensitive to G-CSF, even at lower concentration (10). Mechanistically, SERF1 downregulation led to reduced levels of protein aggregates (Figure 4, D and E). The reduced levels of protein aggregates and aggrephagy were confirmed by a decreased association of mutant NE with BAG3 (Figure 4, F and G) and increased expression of BAG1 and its association with NE (Figure 4, H–K). High-dose G-CSF therapy rescues *ELANE*^EX-3^ missense mutant granulopoiesis through c/EBPβ (2, 10). c/EBPβ is required for ApoE-dependent aggregate formation and aggrephagy in neurodegeneration (31). We hypothesized that aggrephagy activation results in autophagy-induced cell death and its inhibition would allow G-CSF–dependent c/EBPβ rescue of granulopoiesis. We found that silencing of SERF1 shifted the balance from G-CSF–resistant aggrephagy toward G-CSF–sensitive proteasome-mediated clearance of misfolded proteins. These results illustrate protein aggregation–mediated pathobiology of a hematopoietic disorder (Supplemental Figure 12F) and provide support to the notion of targeting aggrephagy pathways as an alternate therapeutic option for G-CSF–resistant SCN.

## Discussion

SCN is frequently associated with dominant point mutations in *ELANE*, encoding NE. *ELANE*^mut^ neutrophil precursors show aberrant distribution of NE, with accumulation in the ER and intracellular juxtamembrane regions. Unfortunately, animal modeling of *ELANE*^mut^ has been hampered by the fact that knockin mice fail to phenocopy human disease (11, 32).

Our understanding of the pathobiology of *ELANE*^mut^ SCN has advanced in the last 10 years using gene-edited human stem cell–derived granulopoiesis (10, 13, 33). iPSCs derived from patients and healthy donors have been used to decipher pathogenetic mechanisms and for drug discovery (34–36). Others and we have used iPSCs to unravel mechanisms of SCN pathogenesis (10, 12, 13, 37, 38). The generation and analysis of hematopoiesis derived from iPSC lines afford the efficient use of high-fidelity tools of gene editing, resulting in bona fide generation and repair of missense mutations through the preferential use of HDR recombination (10). Using CRISPR/Cas9 gene-edited iPSC lines, our group earlier reported that *ELANE* missense mutations are necessary and sufficient to induce SCN pathogenesis (10). We also demonstrated that missense mutations in exon 3 of *ELANE* induce neutropenia that can be ameliorated by G-CSF through alternative activation of *CEBPB*-dependent granulopoiesis, as shown in primary myelopoiesis from patients (39, 40). Our group has previously demonstrated that mutations in the translation initiation codon of *ELANE*, a mutation associated with resistance to G-CSF administration (6–8), result in neutropenia, through the expression of alternative, truncated, misfolded peptides by cistrons initiated by alternative translation start codons and internal ribosomal entry sites (12). In this study, we confirmed that *ELANE* start codon–mutated neutrophil precursors express N-terminally truncated alternate NE and found that they are specifically rich in cytosolic NE aggregates. Unlike G-CSF–sensitive *ELANE* mutations that induce ER and UPR stress (10), we found that the mutation of the *ELANE* translation initiation codon resulted in an alternative proapoptotic pathway. This alternative pathway is the macroautophagic process of aggrephagy. While *ELANE* translation initiation codon mutations did not result in a significant ER/UPR stress response and resulted in predominant nuclear localization of NE, we found downregulated BAG1 expression, increased BAG3/BAG1 ratio, colocalization of NE aggregates in the cytoplasm with BAG3, and localized expression of autophagic LC3B. BAG1 and BAG3 act as co-chaperones (18, 19) driving, respectively, the misfolded protein response toward proteasome-mediated degradation (20) or the macroautophagic process of aggrephagy (15, 21). The existence of larger misfolded NE peptides in *ELANE* translation initiation mutations is the basis of the preferential defect. Our data clearly support the notion that alternate, truncated NE protein induces the aggrephagy machinery in these cells, a mechanism similar to those resulting in neural and glial apoptosis in neurodegenerative diseases (24).

After ruling out a differential expression of HSP70 chaperone family proteins, we found that RNA-chaperone protein SERF1, known to induce misfolded protein aggregates in neurodegenerative diseases, is upregulated in the translation initiation codon *ELANE*^mut^ neutrophil precursors, translocates to cytoplasm, and interacts with the truncated NE protein aggregates. Silencing of SERF1 enhanced survival and differentiation of iPSC-derived neutrophil precursors, rendering them sensitive to G-CSF.

Chronic administration of G-CSF is a first-line treatment of *ELANE*^mut^ SCN. Unfortunately, patients with mutations near the 5′ end of the *ELANE* gene involving the translation initiation codon are resistant to G-CSF administration (6–8), having only HSCT as an available therapy. Given the early age of these patients when receiving HSCT and the large number of comorbidities including bacterial and fungal infections, the mortality of these patients undergoing stem cell transplantation is very high. Chronic G-CSF therapy, even in responding patients, is not without harmful consequences. Up to a quarter of *ELANE*^mut^ SCN cases on long-term, moderate- or high-dose G-CSF therapy develop somatic CSF3R-activating mutations preceding the onset of MDS/AML (9), underscoring the need to investigate alternative therapies that could decrease therapeutic reliance on G-CSF. We have previously demonstrated that the addition of the NE inhibitor sivelestat to G-CSF improves the sensitivity of exon-3 missense mutations of ELANE to G-CSF, aiming to reduce the dose needed to generate granulopoietic responses to G-CSF (10).

In this study, we now demonstrate that interference with the process of aggregation of misfolded truncated NE peptides prevents aggrephagy-induced apoptosis and restores G-CSF sensitivity of otherwise highly resistant neutrophil precursors. Specifically, downregulation of SERF1, a major regulatory chaperone of misfolded protein aggregation and age-related proteotoxicity (26–28) that specifically locates in NE aggregates, completely prevents the need for high-dose G-CSF to induce granulopoiesis.

Our data support the presence of alternative mechanisms of apoptosis that depend on the specific location of each missense mutation. Our work provides an alternate therapeutic option for granulocyte recovery in SCN patients by using lower doses of G-CSF in combination with approaches to downregulate or degrade SERF1, thereby decreasing the risk of these patients to develop secondary MDS and AML.

## Methods

### Sex as a biological variable.

Male- and female-derived iPSCs were used in this study. Sex was not considered as a biological variable since causal heterozygote mutations in *ELANE* equally affect males and females.

### Human iPSC generation from healthy donor (normal) and ELANE translation initiation codon–mutant SCN patient PB MNCs.

iPSC lines, “normal” (from healthy donors) and SCN patients with heterozygous mutation at the *ELANE* translation initiation codon [*ELANE* c.1A>G, p.(M1V) and *ELANE* c.3G>A, p.(M1I)] were generated with lentiviral vectors and characterized as described previously (10, 41). Once derived, iPSC lines were cultured on Matrigel (354277, Corning) and maintained with mTeSR1 medium (85850, StemCell Technologies).

### Cell lines.

RBL-1 cells were obtained from the American Type Culture Collection (ATCC) inventory.

### Gene editing of ELANE translation initiation codon in iPSC lines.

CRISPR/Cas9 gene editing technology was used to correct the mutation (c.1A>G) in the *ELANE* patient iPSC line (GTG-P1) to generate the isogenic *ELANE* mutation–corrected patient iPSC line (GTG-C). Human codon–optimized pX458 Cas9 plasmids were obtained from Addgene and modified by the Cincinnati Children’s Hospital Medical Center Transgenic Core, as previously described (42). The Addgene Zhang’s CRISPR designing tool (https://www.addgene.org/crispr/zhang/) was used to identify a single-guide RNA *ELANE* ATG (Assembly 2) to target *ELANE* exon 1 and cloned into the human codon–optimized pX459 Cas9 plasmid. The asymmetric donor plasmid was designed and synthesized by GenScript. *ELANE* CRISPR and asymmetric donor plasmids were purified. iPSCs were cultured on 6-well Matrigel-coated dishes in mTeSR1 until confluence. Accutase (Sigma-Aldrich) was used to dissociate iPSCs into single cells, and nucleofected with 1 μg each of Assembly 2 CRISPR and 1 μg asymmetric donor construct using kit P3 and the Amaxa Nucleofector (Lonza). Cells were seeded on Matrigel-coated culture dishes after nucleofection, in mTeSR1 medium supplemented with 10 μM Y-27632 (Millipore). Cells were cultured for 2 days, dissociated into single cells using Accutase, and the donor-expressing GFP^+^ iPSCs were sorted (Cincinnati Children’s Flow Sorting facility) and plated onto Matrigel-coated culture dishes in mTeSR1 medium supplemented with 10 μL ROCK inhibitor Y-27632. Selected clones/subclones with the corrected gene edition were confirmed with Sanger sequencing and postediting characterization was assessed.

The healthy donor iPSC line (normal) was gene edited using the CRISPR/Cas9 method to knock in a GTG mutation in place of the translation initiation codon ATG of one allele of the *ELANE* gene to generate isogenic knockin iPSCs (GTG-KI). The same method and procedure that were used for the correction of mutation in the SCN patient–derived iPSCs were followed except for using a different plasmid donor with GTG instead of ATG.

### Hematopoietic and granulopoietic differentiation of healthy donor (normal), SCN patient, and isogenic mutation–knockin and –corrected iPSCs.

Directed hematopoietic differentiation of iPSCs was carried out using a STEMdiff hematopoietic kit (05310, StemCell Technologies) as per manufacturer’s instruction. On day 12, floating CD34^+^CD45^+^ hematopoietic progenitors were harvested and cultured in myeloid expansion medium (IMDM + 10% FBS) containing human SCF (50 ng/mL), human GM-CSF (10 ng/mL), and human IL-3 (10 ng/mL) for 5 days. Cells were counted, analyzed by flow cytometry, and further cultured (5 days) in the presence of G-CSF (50 ng/mL and 1000 ng/mL) for granulopoietic differentiation. At the end of granulopoietic differentiation, cells were evaluated for phenotypic and morphological granulopoietic differentiation using flow cytometry and Wright-Giemsa staining, respectively. The Wright-Giemsa–stained cells were scored morphologically for granulocytic precursor populations of promyelocytes, myelocytes, metamyelocytes, bands and neutrophils, as well as monocytes.

### Flow cytometry, immunophenotypic analysis, and cell sorting.

Further details on antibodies and fluorochrome-conjugated proteins used in this study can be found in Table 1. For the analyses of directed hematopoietic differentiation, floating cells on day 12 of hematopoietic differentiation of iPSCs were harvested, washed and stained for human CD45, CD34, and CD43, and flow analyzed for the hematopoietic progenitors (CD45^+^CD34^+^). Cells on day 12+5 in myeloid culture conditions were analyzed for apoptosis using an annexin V binding assay. Briefly, cells were labeled with antibodies against human CD45, CD34, CD14, CD11b, CD15, and annexin V–APC at dilution of 1:100. Apoptosis of promyelocytes/myelocytes (hCD45^+^hCD34^–^hCD14^–^hCD11b^–^hCD15^+/dim^) was evaluated by analyzing annexin V binding. Cells cultured in granulopoietic differentiation medium containing G-CSF (50 ng/mL and 1000 ng/mL) were flow analyzed for terminal granulopoiesis and monopoiesis (neutrophils: hCD45^+^hCD14^–^hCD11b^+^hCD15^+/hi^hCD66b^+^; monocytes: hCD45^++^hCD14^+^hCD11b^+^). For promyelocyte/myelocyte cell sorting, cells in myeloid culture conditions (day 12+5) were stained for human CD45, CD34, CD14, CD11b, CD15, and promyelocytes/myelocytes (hCD45^+^hCD34^–^hCD14^–^hCD11b^–^hCD15^+/dim^) were FACS isolated, and used for RNA extraction, immunoblots, and confocal immunofluorescence microscopy. A FACSCanto flow cytometer (BD Biosciences) and FACSAria II cell sorter (BD Biosciences) were used for analyses and cell sorting, respectively.

### Quantitative RT-PCR.

Total RNA was extracted from FACS-isolated normal control, GTG-KI, GTG-P1, and GTG-C neutrophil precursors using an RNeasy Mini Kit (74104, QIAGEN) following the manufacturer’s instructions, and cDNA was prepared using TaqMan reverse transcription reagent (N8080234, Applied Biosystems/Life Technologies). The mRNA expression levels of *XBP1s*, *HSPA5*, *ATF6*, and *DDIT3* were analyzed by Q-RT-PCR assay using TaqMan Universal PCR master mix and gene-specific TaqMan primers (Roche Applied Science/Life Technologies). The expression level was normalized to the expression of internal control gene *GAPDH*.

### Immunoblot analyses.

Neutrophil precursors (hCD45^+^hCD34^–^hCD14^–^hCD11b^–^hCD15^+^) were sorted from normal control, GTG-KI, GTG-P1, and GTG-C myeloid cultures. Sorted neutrophil precursors were lysed in RIPA buffer containing protease inhibitor cocktail (04693159001, Roche) and phosphatase inhibitors (046906837001, Roche). The whole-cell lysates were dissolved in 2.1% SDS-containing Laemmli buffer (final concentration of SDS was 1.05%) and boiled for denaturation. Lysates were electrophoresed through 4%–15% SDS-PAGE gradient gels followed by transfer to PVDF membranes. To separate insoluble aggregates and soluble protein fractions, neutrophil precursors were lysed in buffer (50 mM Tris-HCl, pH 8.0, 50 mM NaCl, 5 mM EDTA, 1% Triton X-100 supplemented with protease inhibitor cocktail) for 20 minutes on ice, without SDS. The lysate was cleared by centrifuging at 10,000*g* for 10 minutes at 4°C followed by ultracentrifugation at 200,000*g* for 2 hours. The pellet was resuspended in 100 μL lysis buffer and 100 μL 2× SDS-containing Laemmli buffer. Both pellet and supernatant fractions were processed for SDS-PAGE (4%–15% gradient) and immunoblot analyses. The membranes were probed with primary antibodies against NE from immunized chicken (IgY, dilution 1:5000) (12), BAX, BAK, p-BAD, HSP70, HSP40, HSP90p, SERF1, BAG1, BAG3, and β-actin (dilutions 1:1000) followed by secondary antibodies tagged with HRP (anti–mouse IgG, anti–rabbit IgG, or anti–chicken IgY, dilution 1:5000). The blots were developed using a chemiluminescence-coupled reaction. The band intensities on the x-ray films were quantitated by using ImageJ software (NIH) and normalized to the β-actin band intensity of the corresponding sample.

### Confocal immunofluorescence microscopy.

Sorted neutrophil precursors (hCD45^+^hCD34^–^hCD14^–^hCD11b^–^hCD15^+^) from day 10+5 myeloid culture were fixed (4% paraformaldehyde), permeabilized (0.1% Triton X-100), blocked (3% goat serum or 3% BSA), and treated with primary antibodies against NE (specific for C-terminal region of NE, dilution 1:2000) (12), anti-NE antibodies (specific for N-terminal region of NE, dilution 1:100), anti-LC3B (dilution 1:200), and anti-SERF1 (dilution 1:200) overnight at 4°C. Cells were washed and stained using fluorochrome-tagged secondary antibodies (dilution 1:500), washed, and mounted with ProLong Gold antifade mounting media with DAPI (P36935, Thermo Fisher Scientific) to counterstain the nuclei. For the single-cell quantification of nuclear NE distribution, cells with predominantly nuclear NE distribution were counted as those in which the nuclear mean fluorescence intensity (MFI) per surface area represented more than 50% of the cell NE-associated fluorescence. For the visualization of protein (NE) aggregates, sorted normal control, GTG-KI, GTG-P1, and GTG-C iPSC neutrophil precursors were fixed with 4% paraformaldehyde (15 minutes, room temperature), washed, and permeabilized with PBS supplemented with 0.1% Triton X-100, 0.03% Tween 20, and 1% BSA (15 minutes, room temperature). Cells were washed and blocked with PBS supplemented with 1% BSA and 0.03% Tween 20 (1 hour, room temperature). Cells were treated with anti-NE antibody (chicken IgY at 1:2000 dilution) (12) overnight at 4°C. Cells were washed and incubated with Alexa Fluor 488–conjugated donkey anti-chicken IgY (1:500) and ProteoStat (Enzo, 1:2500) for 1 hour at room temperature. Cells were washed, mounted with ProLong Gold antifade mounting media containing 1 μg/mL DAPI, and were analyzed using an LSM 710 confocal system (Zeiss) attached to an inverted microscope (Observer Z1, Zeiss) equipped with a Plan Apochromat ×63 1.4 NA oil immersion lens.

### PLA and confocal microscopy.

PLA was performed to detect interactions between proteins residing in close proximity or in a multiprotein complex, as per the manufacturer’s instructions (DUO92002 and DUO92004, Sigma-Aldrich). Briefly, normal control, GTG-KI, GTG-P1, and GTG-C neutrophil precursors were cytospun onto Superfrost Plus microscope slides (Thermo Fisher Scientific), fixed using 4% paraformaldehyde, permeabilized with 0.1% Triton X-100 for 10 minutes, followed by blocking with DuoLink Blocking solution. Cells were treated with primary antibodies against SERF1 and NE or BAG3 and NE or BAG1 and NE or AAT and NE or AAT and SERF1 overnight at 4°C. Cells were washed and treated with rabbit and mouse secondary antibodies coupled with nucleotide probes (DuoLink InSitu PLA probe α-Rabbit PLUS and DuoLink InSitu PLA probe α-Mouse MINUS or DuoLink InSitu PLA probe α-Rabbit PLUS and DuoLink InSitu PLA probe α-Goat MINUS) for 1 hour at 37°C. The probes were ligated together (30 minutes at 37°C), hybridized to form a circular DNA. Fluorescently labeled oligonucleotides were then incorporated during the process of rolling-circle amplification (100 minutes at 37°C). Cells were washed and mounted using ProLong Gold antifade mounting media containing DAPI. The stained cells were analyzed using a Zeiss LSM 710 confocal system attached to an inverted microscope (Observer Z1, Zeiss) equipped with a Plan Apochromat ×63 1.4 NA oil immersion lens.

### Statistics.

Data are presented as individual data and mean ± standard deviation of a minimum of 3 replicates per experiment and a minimum of 2 independent experiments. Statistically significant differences were assessed by Student’s *t* test for 2 independent variables or for analyses of more than 2 variables, by 1-way or 2-way ANOVA tests with Bonferroni’s correction.

### Study approval.

PB from healthy donors and SCN patients carrying the *ELANE* start codon mutation (*ELANE* c.1A>G, *ELANE* c.3G>A) was obtained at Cincinnati Children’s Hospital Medical Center through informed consent under an approved institutional review board research protocol.

### Data availability.

Raw data are provided in the supplemental Supporting Data Values file. The authors declare that all data supporting the findings of this study are available, to the best of our effort, within this paper and supplemental information files, and will be available from the corresponding authors upon request. Human subject data will be available deidentified.

## Author contributions

RCN, SE, LRT, AMW, AKS, and BK performed experiments. KCM and TAK generated clinical data. RCN, CML, and JAC supervised the project and designed experiments. MSH generated reagents specifically for this study and generated clinical data. RCN and JAC interpreted data and wrote the manuscript. All authors contributed intellectually to the project and reviewed the manuscript.

## Supplementary Material

Supplemental data

Unedited blot and gel images

Supporting data values

## Figures and Tables

**Table 1:**
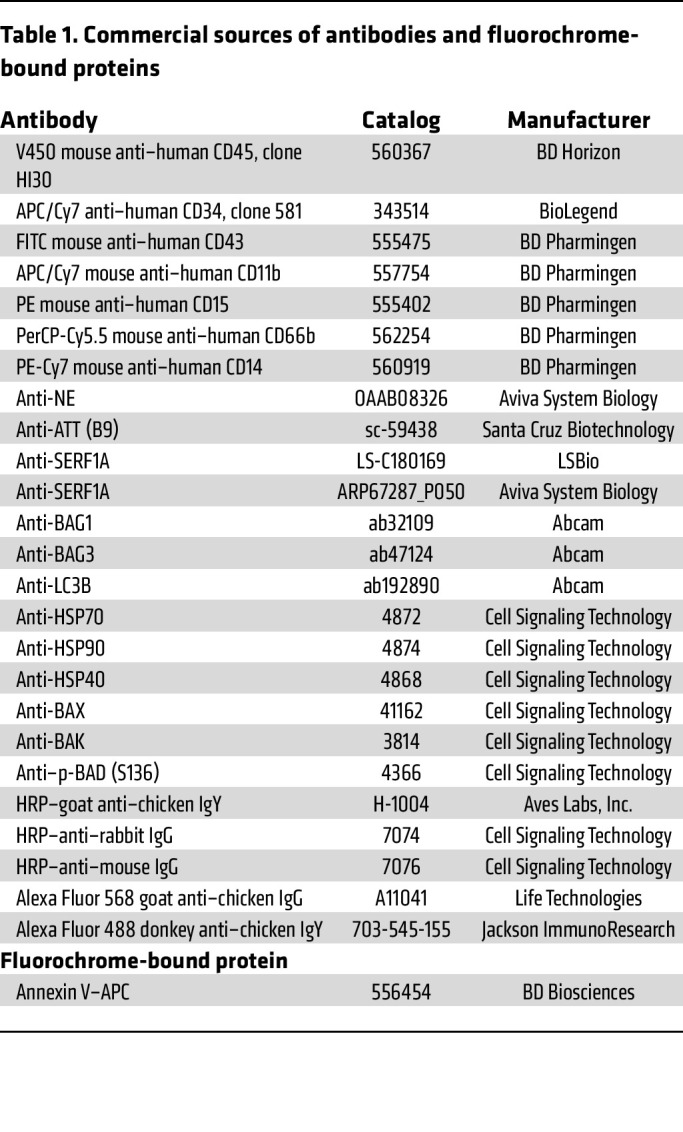
Commercial sources of antibodies and fluorochrome-bound proteins

## Version 1 11/19/2024

In-Press Preview

## Version 2 01/16/2025

Electronic publication
