# Supplementary material for: G-CSF resistance of ELANE-mutant neutropenia depends on SERF1-containing truncated–neutrophil elastase aggregates
Source: J Clin Invest. 2024 Nov 19;135(2):e177342. doi: 10.1172/JCI177342 (PMC11735094; doi:10.1172/JCI177342)

Supplementary Figures

Supplementary figure 1. Characterization of gene-edited isogenic iPSC lines. A) Depiction of 4 iPSC lines used in the study: Normal (healthy donor); isogenic gene edited GTG-KI (GTG knock-in in place of translation initiation codon ATG in one allele of *ELANE* gene in the healthy donor normal iPSC line); SCN patient derived GTG-P1 (*ELANE* gene translation initiation codon mutation c.1A>G); isogenic mutation corrected GTG-C (Correction of mutation in GTG-P1 iPSC). B) Chromatogram map showing no mutation in healthy donor normal iPSC line, presence of *ELANE* translation initiation codon mutation (c.1A>G) on one allele in GTG-P1 patient iPSC line, introduction of GTG in place of ATG on one allele of *ELANE* gene in normal iPSC line and correction of GTG to ATG in patient iPSC line. C) Flow cytometry histogram plots showing expression of pluripotency markers (OCT3/4, SSEA4, Tra-1-60, Tra-1-81, and CD9) in all four iPSC lines. D) All four iPSC lines show normal karyotypes after 10 passages.

Supplementary figure 2. Hematopoietic differentiation of healthy donor and *ELANE* start codon mutant iPSCs. A) FACS dot plots showing hematopoietic progenitors ($CD45^{+}CD34^{+}$) derived from directed differentiation of normal, GTG-KI, GTG-P1 and GTG-C iPSC lines. B) Quantifications of $CD45^{+}CD34^{+}$ hematopoietic progenitors at day 12 of directed hematopoietic differentiation of normal, GTG-KI, GTG-P1 and GTG-C iPSC lines. Over 80% of hematopoietic progenitors are $CD43^{+}$. Comparison between groups from more than 3 independent experiments was calculated using one way ANOVA. N.S.: not significant.

Supplementary figure 3. *ELANE* translation initiation codon mutant neutrophil precursors show increased cell death, impaired neutrophil maturation, and are resistant to high-dose G-CSF. A) Phase contrast images of cell culture of normal, GTG-KI, GTG-P1 and GTG-C

iPSC derived hematopoietic progenitors in myeloid expansion culture condition containing SCF, Il-3 and GM-CSF (O.M. 4x). B) FACS dot plots showing annexin V binding of neutrophil precursors ($CD45^{+}CD34^{-}CD11b^{-}CD15^{+}$) derived from myeloid expansion culture of normal, GTG-KI, GTG-P1 and GTG-C iPSC derived hematopoietic progenitors. C-D) Flow cytometry analyses of the granulopoietic differentiation ($CD45^{+}CD14^{-}CD11b^{+}CD15^{+}CD66b^{+}$ percentage) of normal, GTG-KI, GTG-P1 and GTG-C iPSC derived hematopoietic progenitors in presence of 50 ng/ml (B) and 1000 ng/ml (C) G-CSF. E) FACS dot plots showing mature neutrophil population ($CD14^{-}CD11b^{+}CD15^{+}$). F) Differential counts (%) of the granulopoietic differentiation (at 1000 ng/ml G-CSF) of normal, GTG-KI, GTG-P1 and GTG-C iPSC derived hematopoietic progenitors. G) Representative morphological microphotographs of output cells of G-CSF (1000 ng/mL) induced differentiation of normal, GTG-KI, GTG-P1 and GTG-C iPSC derived hematopoietic progenitors (Wright-Giemsa staining; O.M. 40x). bar=10 μm H) Mean fluorescence intensities of CSF3R expression on cell surfaces of healthy donor and translation initiation codon mutation neutrophil precursors. Comparison between groups from a minimum of 3 independent experiments was calculated using one way ANOVA. * $p<0.05$; ** p,0.01; *** $p<0.001$.

Supplementary figure 4. Hematopoietic differentiation of additional clones of healthy donor and *ELANE* start codon mutant iPSCs. A) FACS dot plots showing hematopoietic progenitors ($CD45^{+}CD34^{+}$) derived from directed differentiation of normal (28l cl 5, 35L cl 6) GTG-KI (cl 5, cl 17), GTG-P1 (cl 5, cl 8) and ATG-C (cl 24.1, cl 24.3) iPSC lines. B) Quantifications of $CD45^{+}CD34^{+}$ hematopoietic progenitors at day 12 of directed hematopoietic differentiation of normal, GTG-KI, GTG-P1 and GTG-C iPSC lines. Over 80% of hematopoietic progenitors are $CD43^{+}$. Data from 2 independent experiments of hematopoietic differentiation was presented.

Supplementary figure 5. ***ELANE*** **translation initiation codon mutant neutrophil precursors show increased cell death, impaired neutrophil maturation, and are resistant to high-dose G-CSF.** A) Phase contrast images of cell culture of normal (28L cl 5, 35L cl6), GTG-KI (cl 5, cl 17), GTG-P1 (cl 5, cl 8) and GTG-C (cl 24.1, cl 24.3) iPSC derived hematopoietic progenitors in myeloid expansion culture condition containing SCF, Il-3 and GM-CSF (O.M. 4x). B-C) FACS dot plots (B) and quantification (C) of annexin V binding of neutrophil precursors ($CD45^{+}CD34^{-}CD11b^{-}CD15^{+}$) derived from myeloid expansion culture. D-E) Flow cytometry analyses of the granulopoietic differentiation ($CD45^{+}CD14^{-}CD11b^{+}CD15^{+}CD66b^{+}$ percentage) of additional clones of normal, GTG-KI, GTG-P1 and GTG-C iPSC derived hematopoietic progenitors in presence of 50 ng/ml, F-G) Granulocyte maturation at 1000 ng/ml G-CSF. Comparison between groups from 2 independent experiments in duplicates or triplicates was calculated using one way ANOVA. * p<0.05; ** p,0.01; *** p<0.001. N.S.: not significant.

Supplementary figure 6. ***ELANE*** **translation initiation codon mutant neutrophil precursors show impaired neutrophil maturation**. A) Differential counts (%) of the granulopoietic differentiation of 28L cl 5, GTG-KI cl5, GTG-P1 cl 5 and GTG-C cl 24.1 iPSC derived hematopoietic progenitors. B) Representative morphological microphotographs of output cells of G-CSF (50 ng/mL) induced differentiation (Wright-Giemsa staining; O.M. 40x). C) Differential counts (%) of the granulopoietic differentiation of 35L cl 6, GTG-KI cl 17, GTG-P1 cl 8 and GTG-C cl 24.3 iPSC derived hematopoietic progenitors. D) Representative morphological microphotographs of output cells (Wright-Giemsa staining; O.M. 40x). bar=10 μm Comparison between groups from 2 independent experiments in duplicates was calculated using one way ANOVA. * p<0.05; ** p,0.01; *** p<0.001.

Supplementary figure 7. Anti-NE C-terminus antibody is specific for the C-terminus domain of NE, and translation initiation codon mutation led to nuclear localization of mutant NE. A) Representative confocal immunofluorescence images of RBL-1 cells expressing NE FL (full length), NE ΔC-terminus (ΔC225-H238) using antibodies specific to NE C-terminus regions. B) Western blots of NE FL (full length), NE ΔC-terminus using antibodies specific to NE C-terminus regions. C) Confocal immunofluorescence images of NE using anti-C-terminus antibody in normal, GTG-KI, GTG-P1 and GTG-C granulocyte precursors. D) Quantification of the percentage of cells with predominant nuclear distribution of NE. A total of 8-10 fields of images in 2-3 independent experiments were evaluated. bar=10 μm. Comparison between groups was calculated using one way ANOVA. * p,0.05; ** p<0.01. N.S.: not significant.

Supplementary figure 8. *ELANE* translation initiation codon mutation results in the expression of N-terminal truncated alternate NE peptide and aggregated NE. A-B) Representative confocal microscopic images (A) and mean fluorescence intensities (B) of NE expression using α–NE Ab against N-terminal region of the protein. *ELANE* translation initiation codon mutation neutrophil precursors express significantly reduced levels of normal NE. bar=10 μm. C) Western blots of NE and β-ACTIN in healthy donor (normal) and ATA-P2 derived iPSC line with a translation start codon mutation showing neutrophil precursors with alternate NE expression. GTG-P2 cell lysate was run along with cell lysates in Figure 2A. D) Western blots showing no alternate or aggregated NE expression in healthy donors and $ELANE^{EX\text{-}3}$ mutant (I118N, Q96P) iPSC derived neutrophil precursors. The mean fluorescence intensity of more than 10 cells from 2 independent experiments was quantified. Comparison between groups was calculated using one way ANOVA. ** p<0.01, *** p<0.001. N.S.: not significant.

Supplementary figure 9. *ELANE* start codon mutant neutrophil precursors show NE aggregation. A) Representative confocal microscopic images of NE in association with ProteoStat fluorescent molecular rotor dye that fluoresce upon interaction with misfolded and aggregated proteins of healthy donor (28l cl 5, 35L cl 6) GTG-KI (cl 5, cl 17), GTG-P1 (cl 5, cl 8) and ATG-C (cl 24.1, cl 24.3) iPSC line derived neutrophil precursors. *ELANE* translation initiation codon mutant cells show high levels of ProteoStat bound aggresome in association with alternate NE. bar=10 μm B) Mean fluorescence intensities (MFI) of ProteoStat stained aggresome. The MFI of more than 25 cells from 2 independent experiments was quantified. Comparison between groups was calculated using one way ANOVA. * $p<0.05$. N.S.: not significant.

Supplementary figure 10. *ELANE* start codon mutant neutrophil precursors show increased levels of aggrephagy process. A) Representative western blot of BH3-only containing pro-apoptotic proteins p-BAD, BAX and BAK in normal, GTG-KI, GTG-P1 and GTG-C neutrophil precursors. The membrane in Figure 2A was used to probe p-BAD, BAX and BAK. B) Quantitative real time polymerase chain reaction (Q-RT-PCR) analyses of the expression (mRNA) ER-stress/UPR pathway genes *XBP1, HSPA5, ATF6, DDIT3*. C-D) Representative confocal microscopic images (C) and mean fluorescence intensities (D) of LC3B in normal, GTG-KI, GTG-P1 and GTG-C neutrophil precursors. The MFI of more than 15 cells from 2 independent experiments was quantified. bar=10 μm. Comparison between groups were calculated using one way ANOVA. ** $p<0.01$, *** $p<0.001$. N.S.: not significant.

Supplementary figure 11. *ELANE* start codon mutant neutrophil precursors show no changes in expression of chaperone proteins that regulate protein folding to native state. A) Representative immunoblots of chaperone proteins HSP90, HSP70, HSP40 in normal, GTG-KI, GTG-P1 and GTG-C iPSC derived neutrophil precursors. No change was observed in the expressions of these chaperones that are known to regulate protein folding to native state. B) Representative confocal microscopic images of PLA signal between NE and α1-anti trypsin (AAT) showing NE interaction with AAT. C) Representative confocal microscopic images of PLA signal between SERF1 and α1-anti trypsin (AAT) showing no interaction. bar=10 μm. A minimum of 2 independent experiments were performed.

Supplementary figure 12. SERF1 downregulation in HL60 and iPSC derived neutrophil precursor cells. A-B) Representative confocal microscopic images (A) and mean fluorescence intensities (B) showing down regulation of SERF1 expression in SERF1 shRNA (#1 and #2) transduced HL60 cells. C-D) Representative confocal microscopic images (C) and MFI (D) of SERF1 expression in Ntg and SERF1 shRNA transduced GTG-P1 iPSC derived neutrophil precursors. bar=10 μm. The MFI of more than 10 cells from 2 independent experiments was quantified. E) SERF1 downregulation increases cell survival of translation start codon mutant granulocyte precursors, F) Working model of SERF1 mediated aggregation mutant NE and proteo-toxicity in *ELANE* translation initiation mutant SCN. Comparison between groups between groups were calculated using one way ANOVA. ** p,0.01; ** p<0.01.

Supplementary Figure 1

A

iPSC lines	Type
Normal	Healthy donor derived iPSC
GTG-KI	GTG knock-in in place of ATG in one allele of *ELANE* gene in healthy donor iPSC
GTG-P1	SCN patient with ATG to GTG mutation in one allele of *ELANE* gene
GTG-C	Correction of GTG to ATG in GTG-P1 patient iPSC line

B

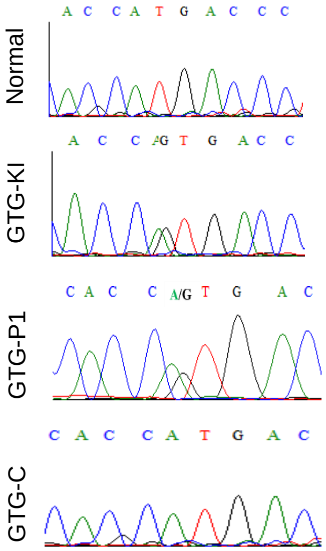

C

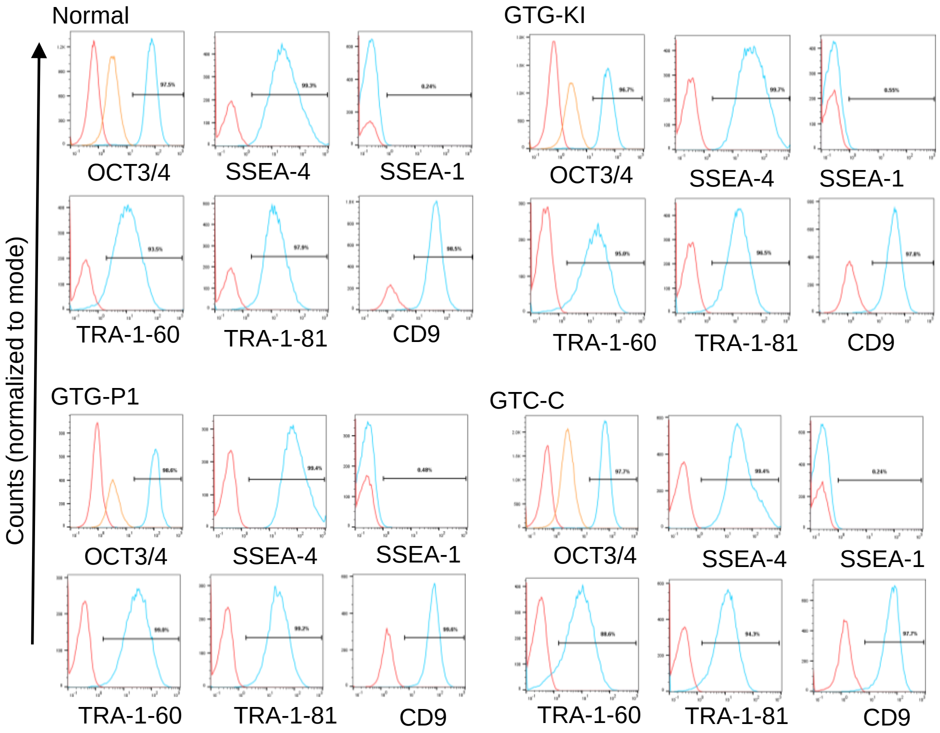

D

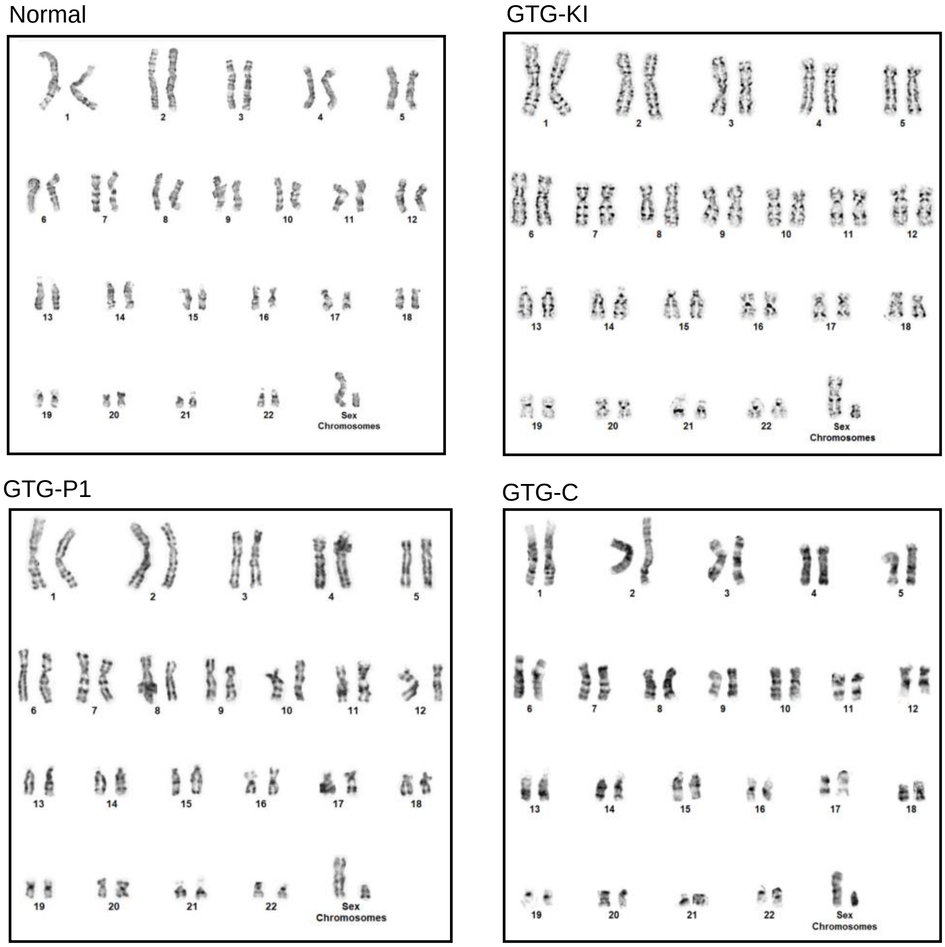

Supplementary Figure 2

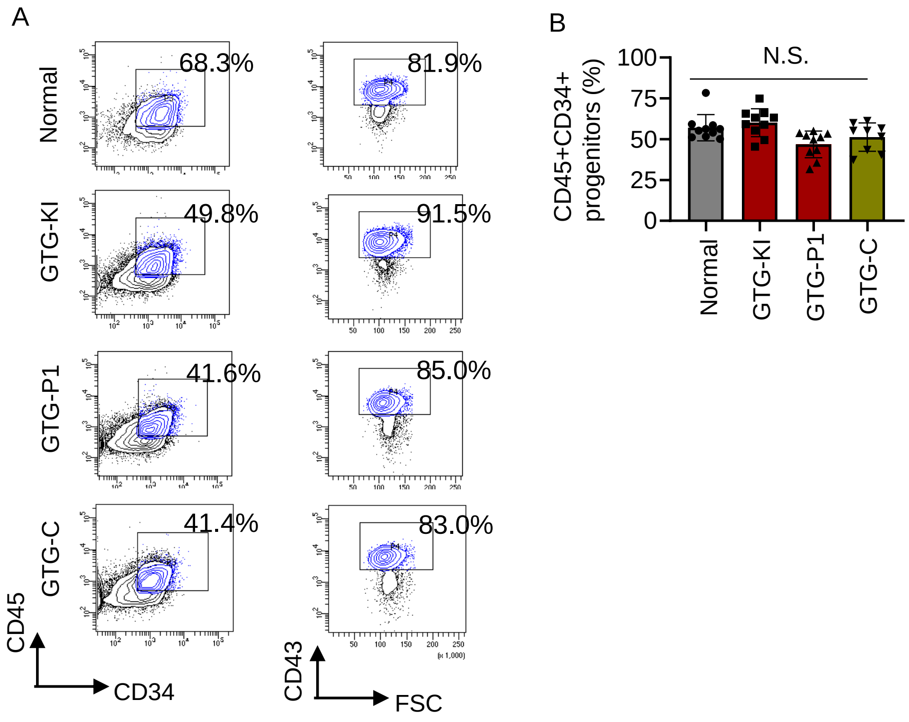

Supplementary Figure 3

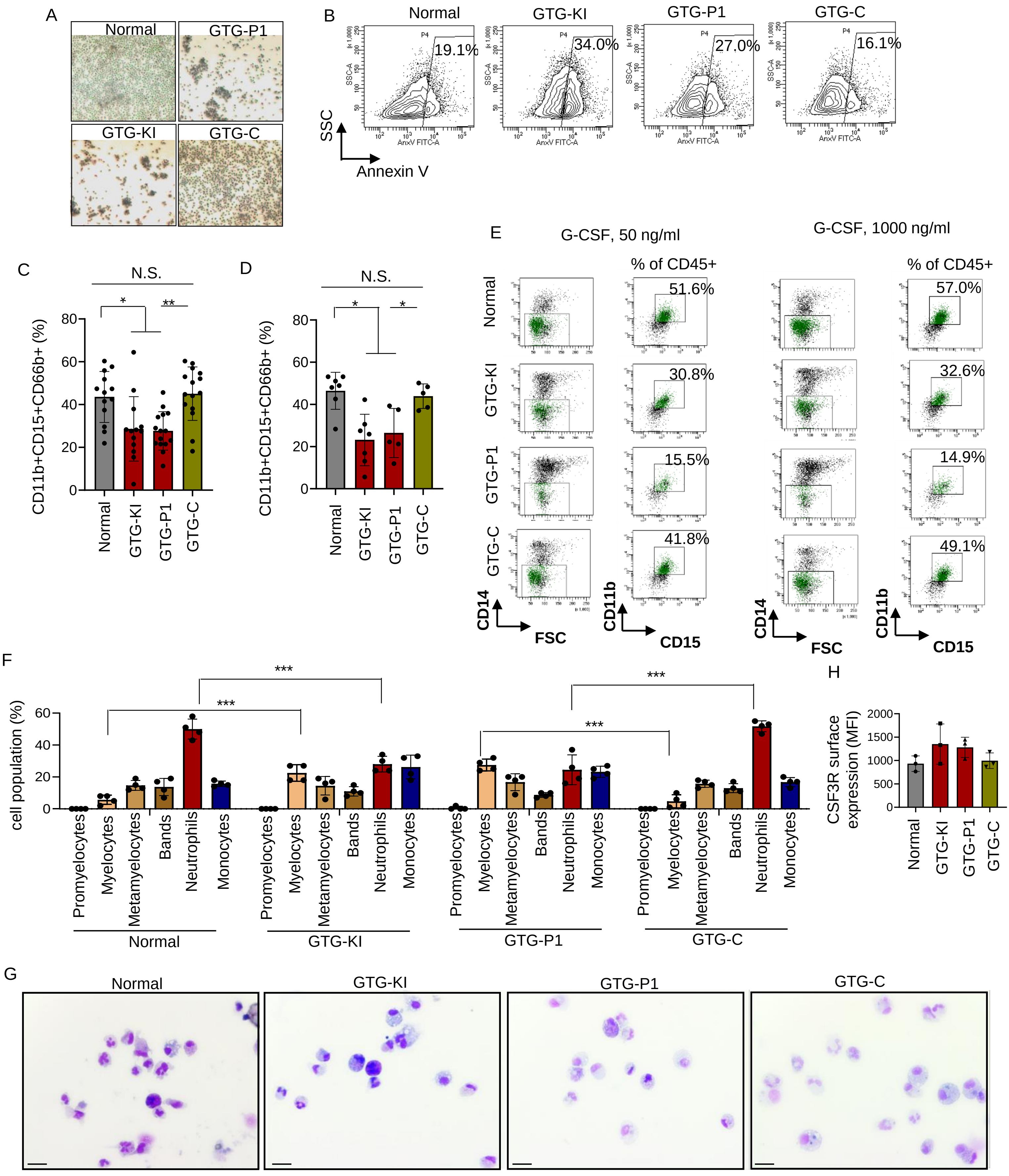

Supplementary Figure 4

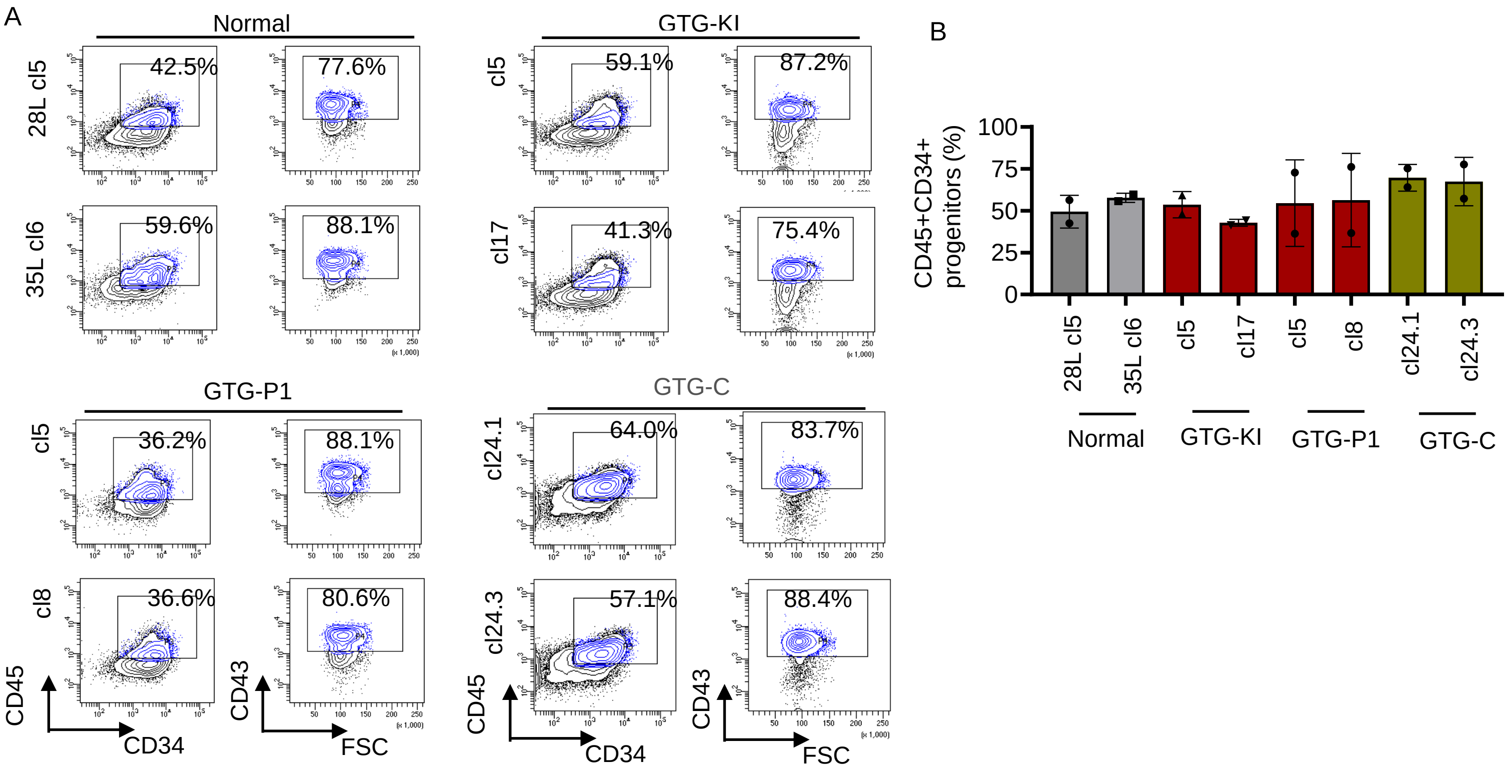

Supplementary Figure 5

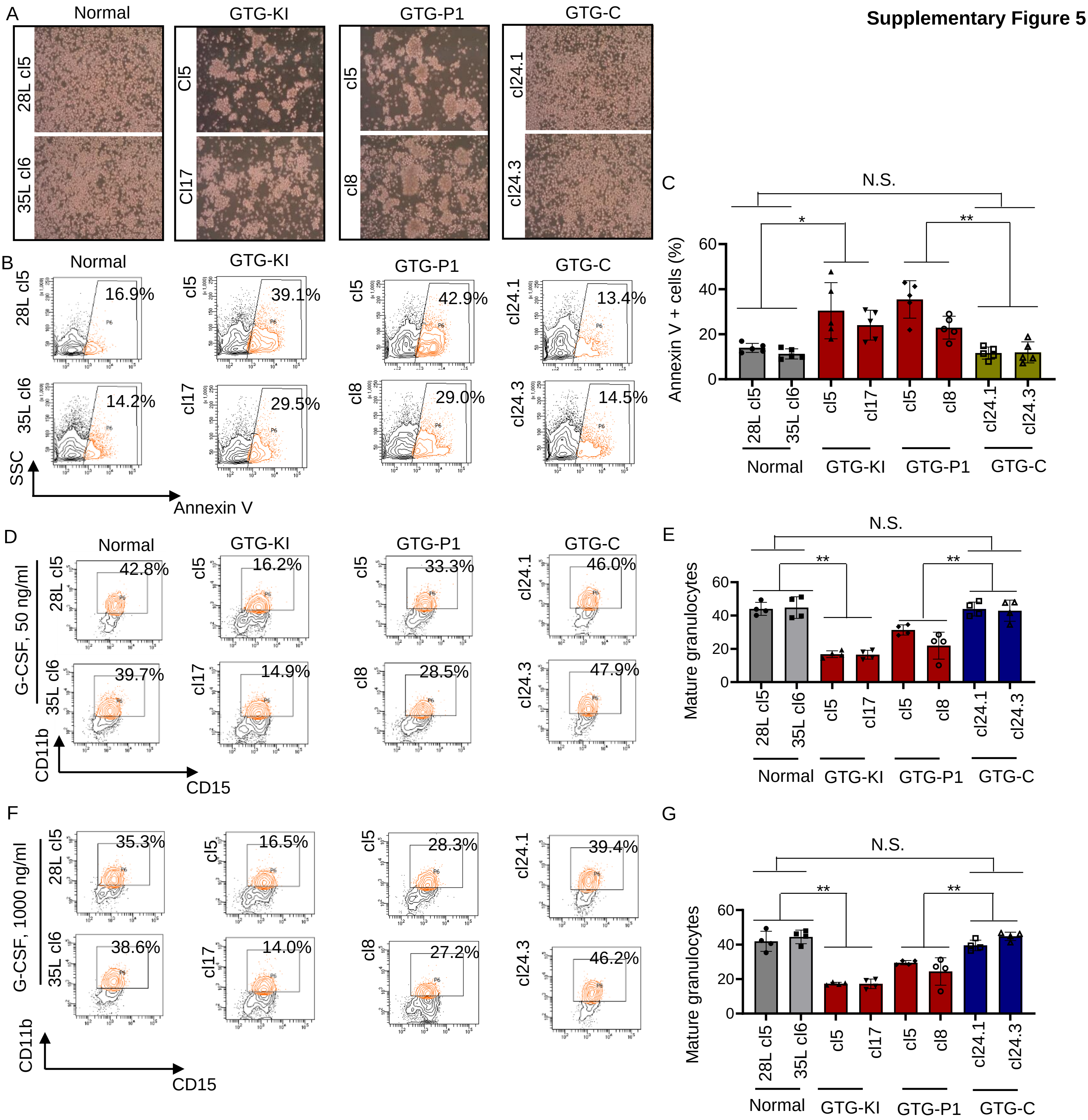

Supplementary Figure 6

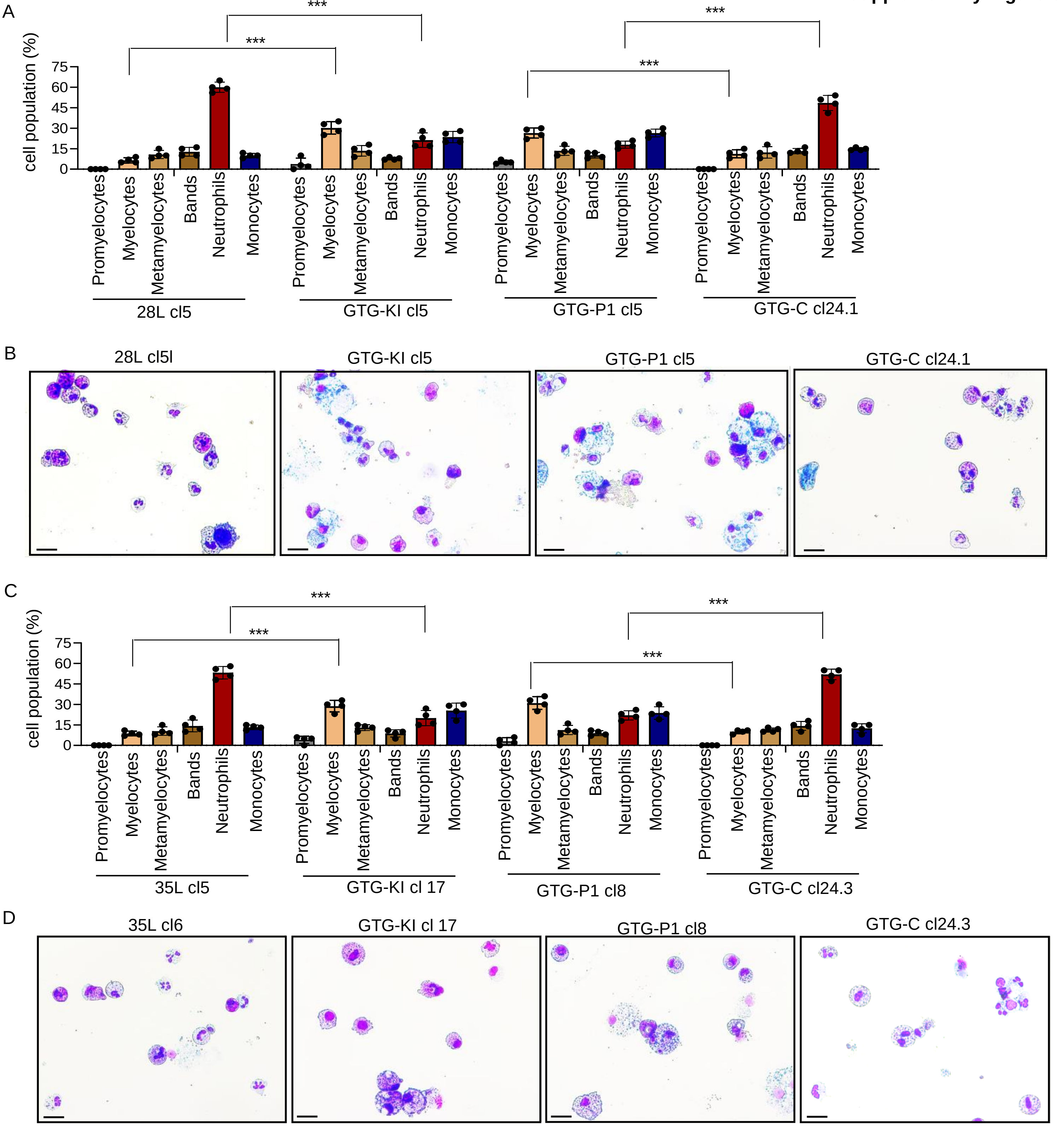

Supplementary Figure 7

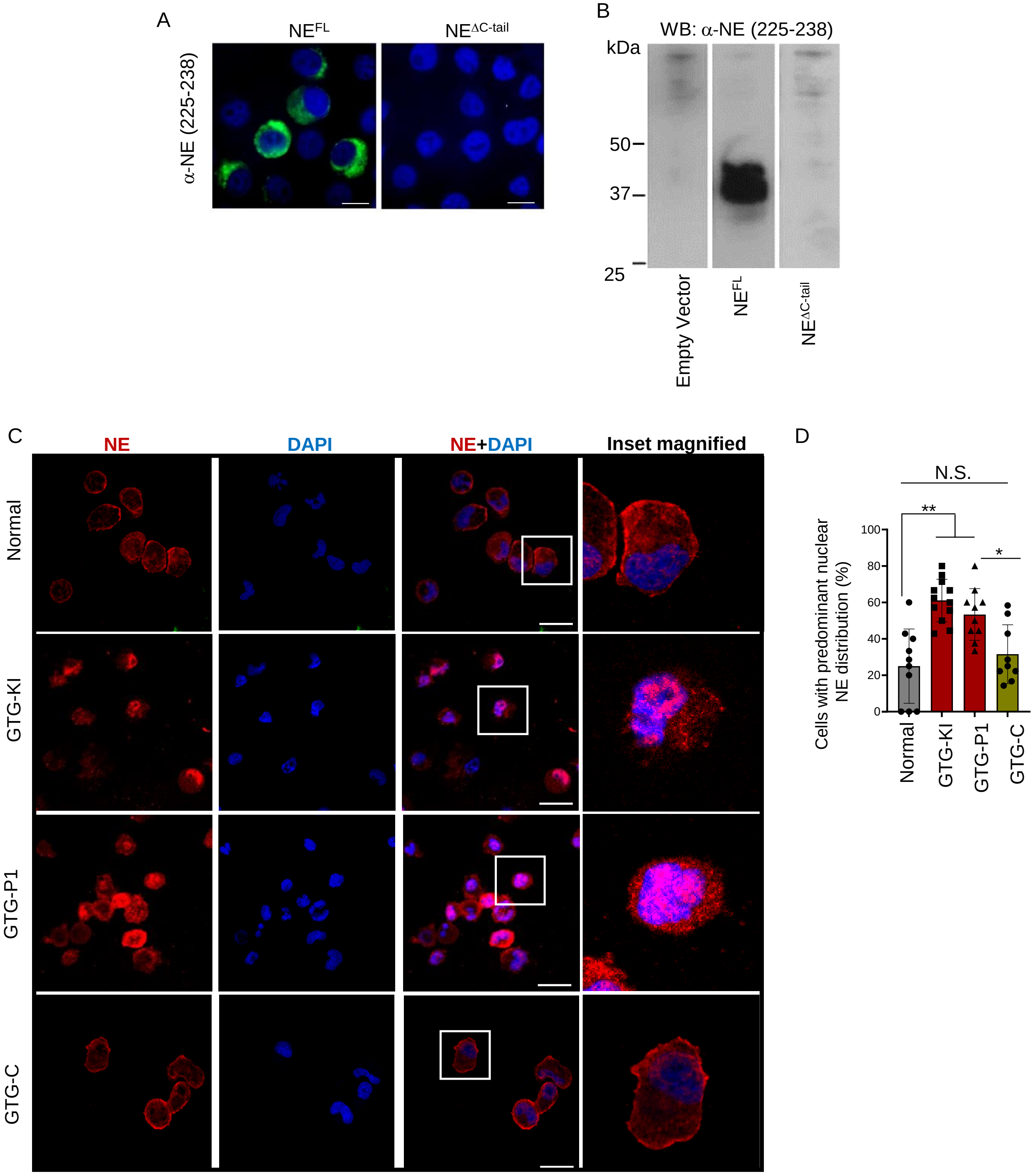

Supplementary Figure 8

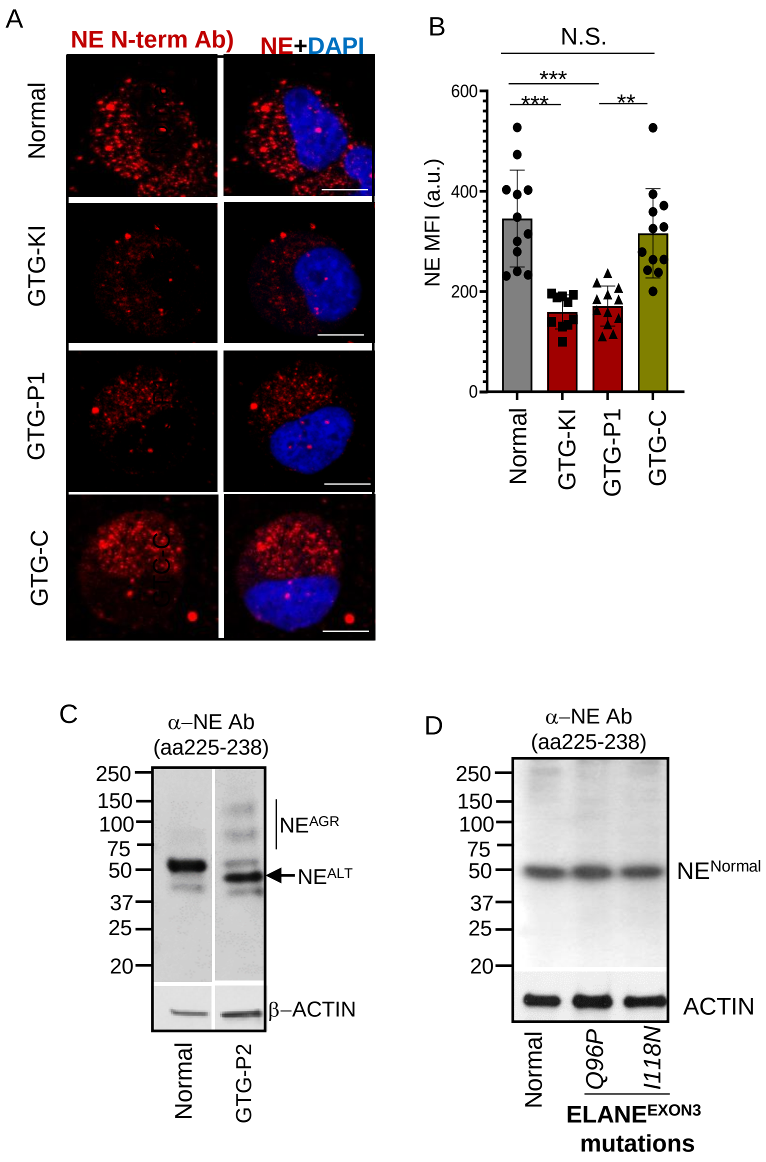

Supplementary Figure 9

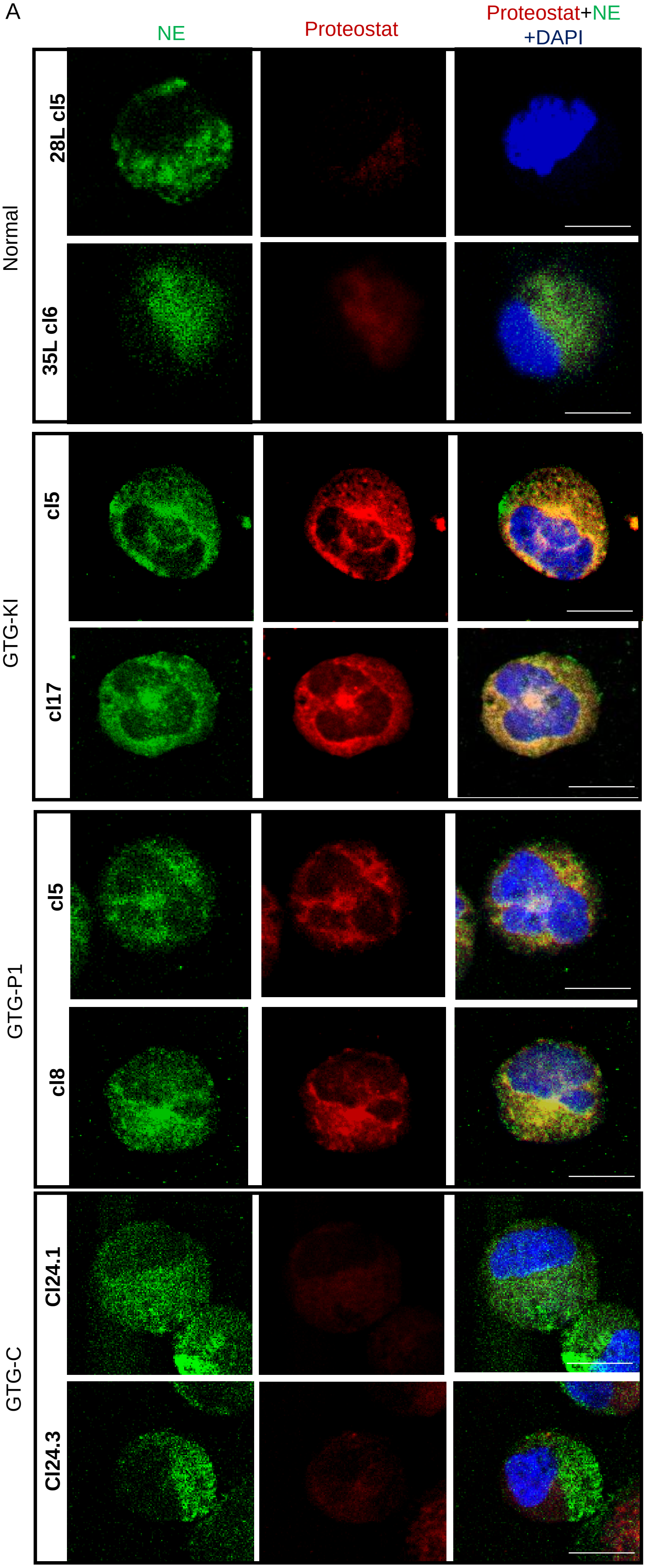

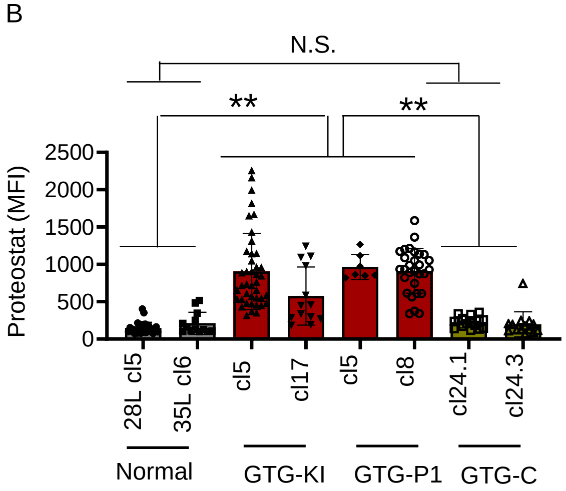

Supplementary Figure 10

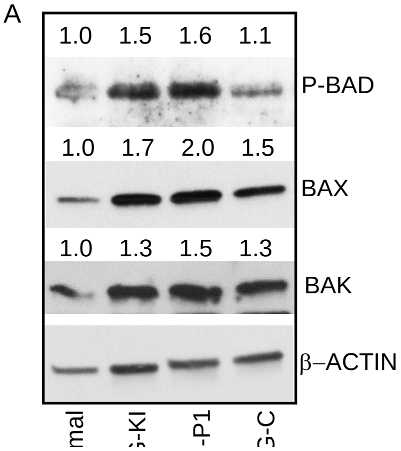

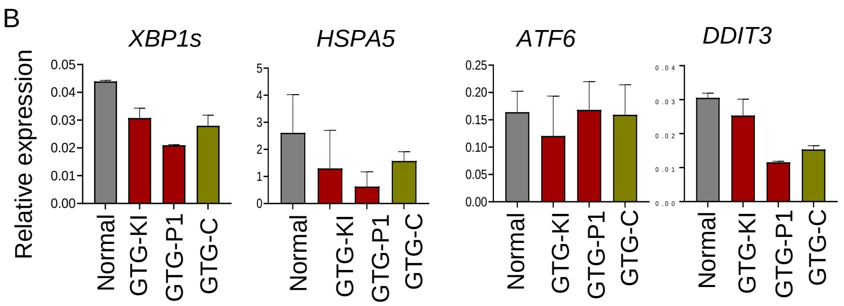

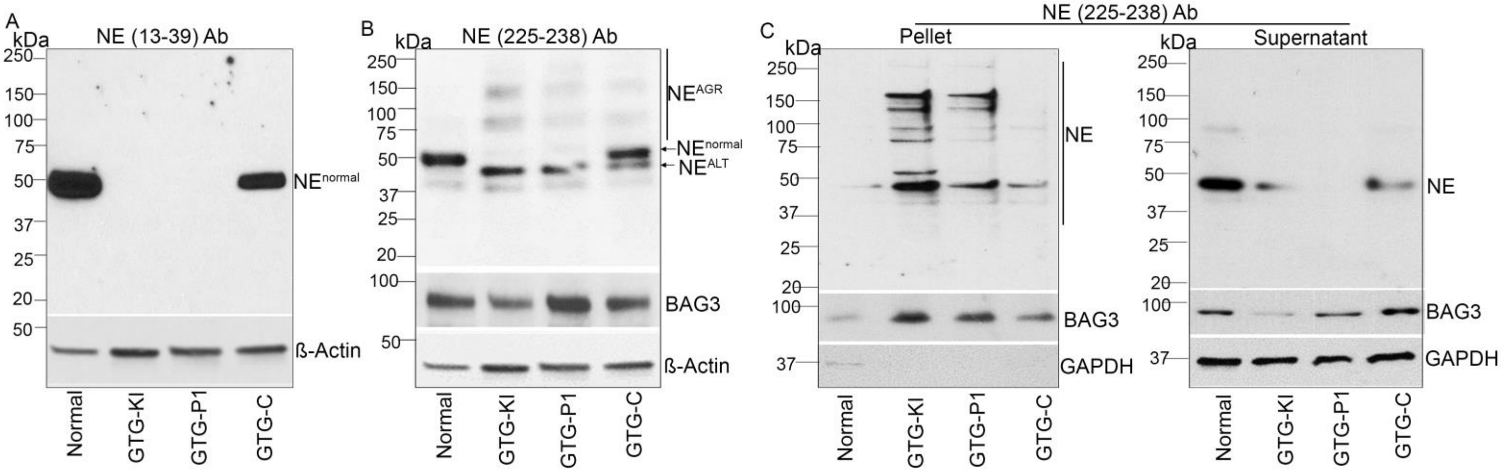

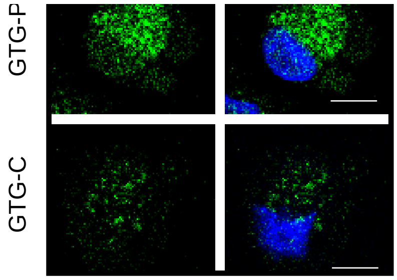

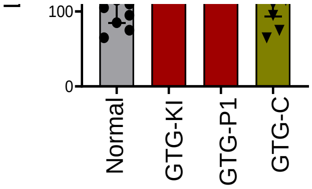

Supplementary Figure 11

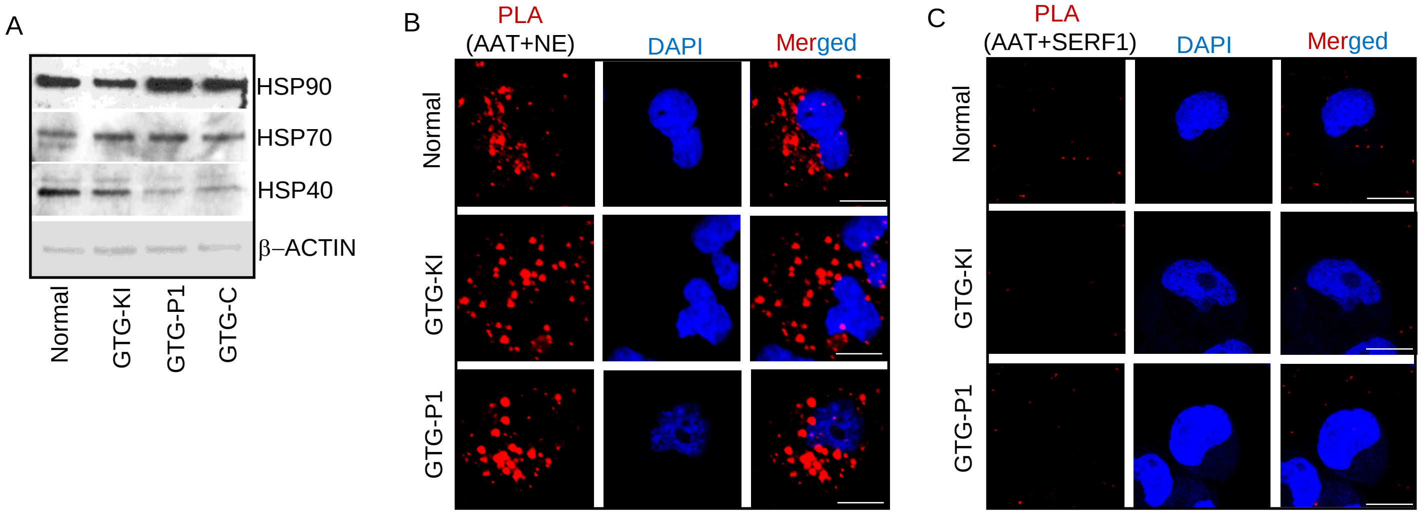

Supplementary Figure 12

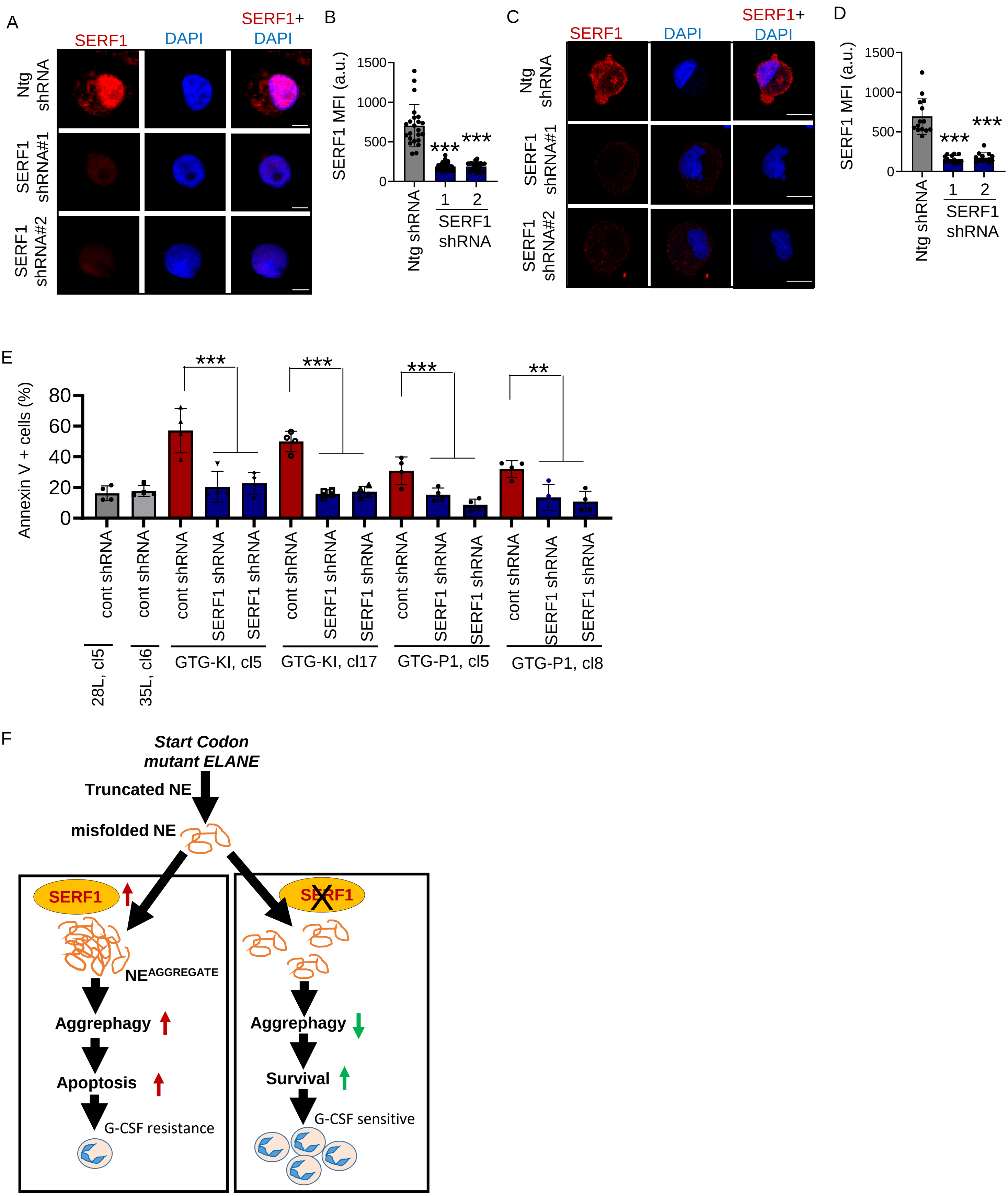

Supplement: Supplemental data [file jci-135-177342-s035.pdf]
