# Supplementary material for: G-CSF resistance of ELANE-mutant neutropenia depends on SERF1-containing truncated–neutrophil elastase aggregates
Source: J Clin Invest. 2024 Nov 19;135(2):e177342. doi: 10.1172/JCI177342 (PMC11735094; doi:10.1172/JCI177342)

Figure 2A

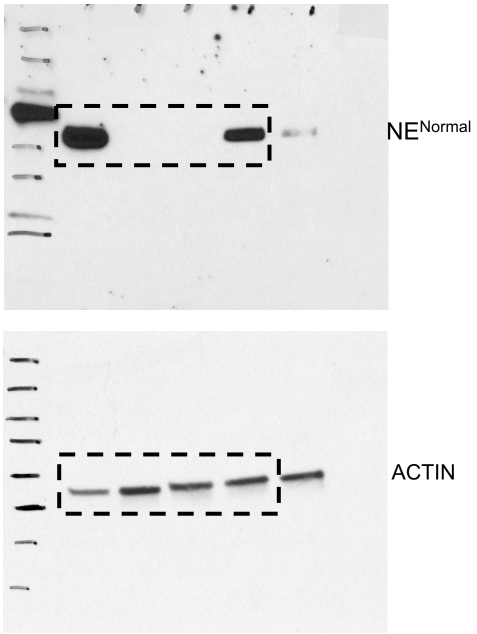

Figure 2B

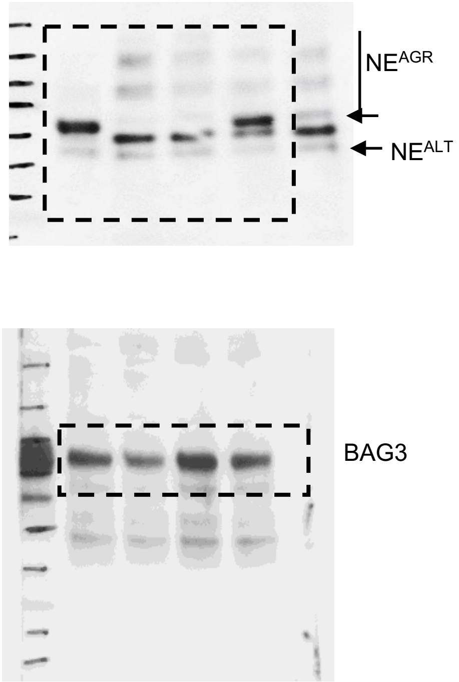

Figure 2I

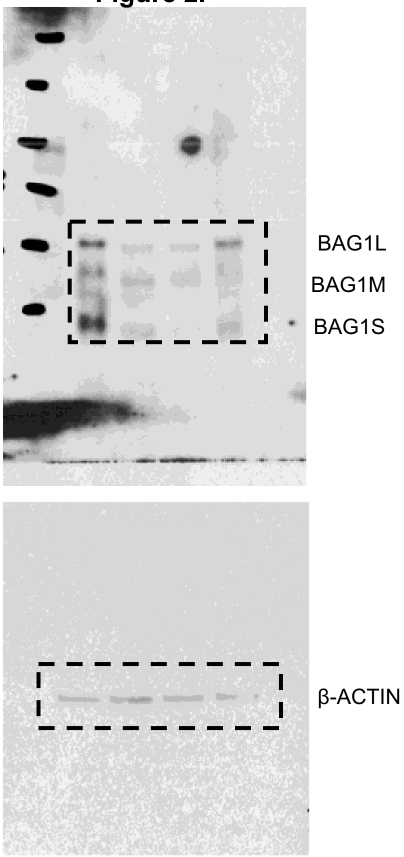

Figure 2C

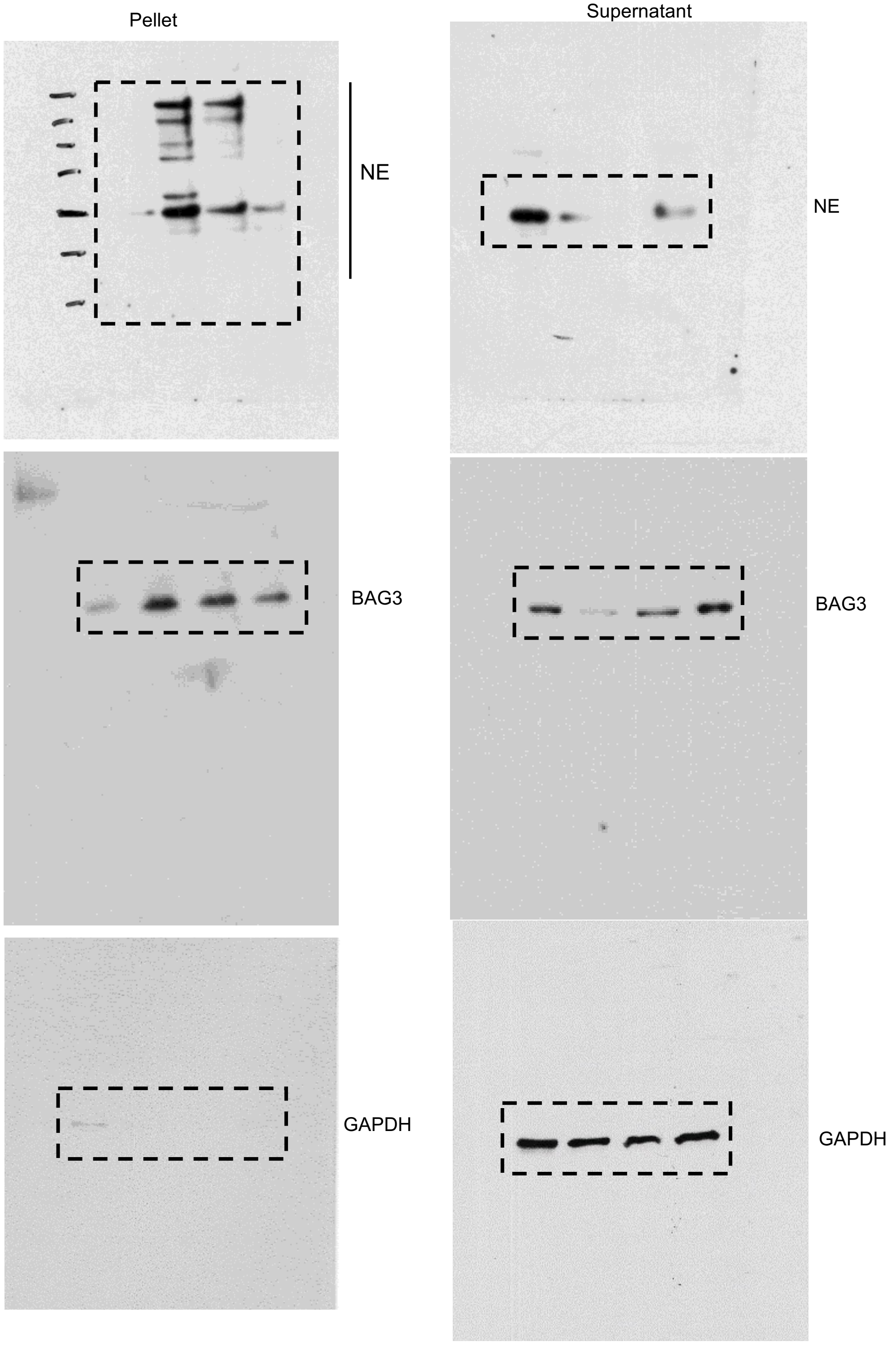

Figure 3A

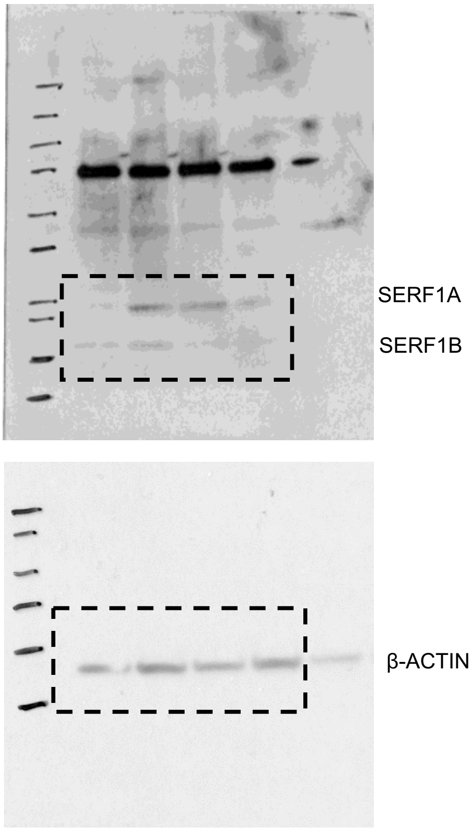

Supplementary Figure 8C

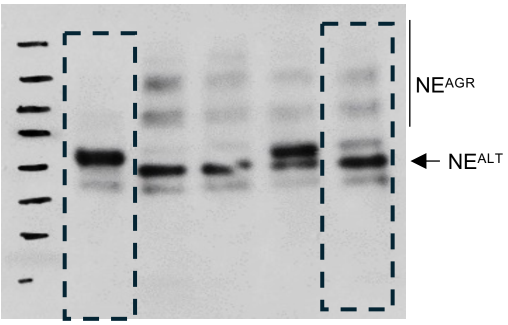

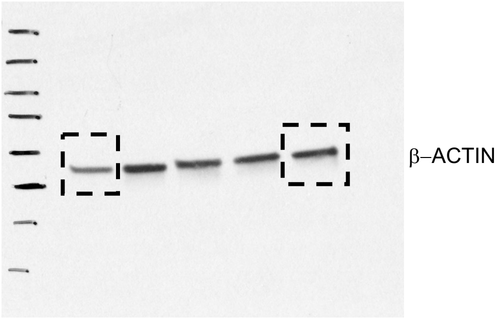

Supplementary Figure 8D

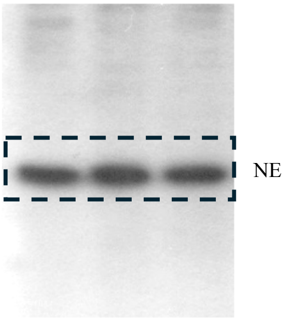

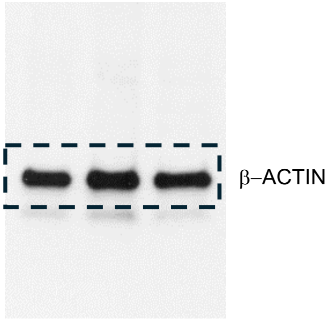

Supplementary Figure 10A

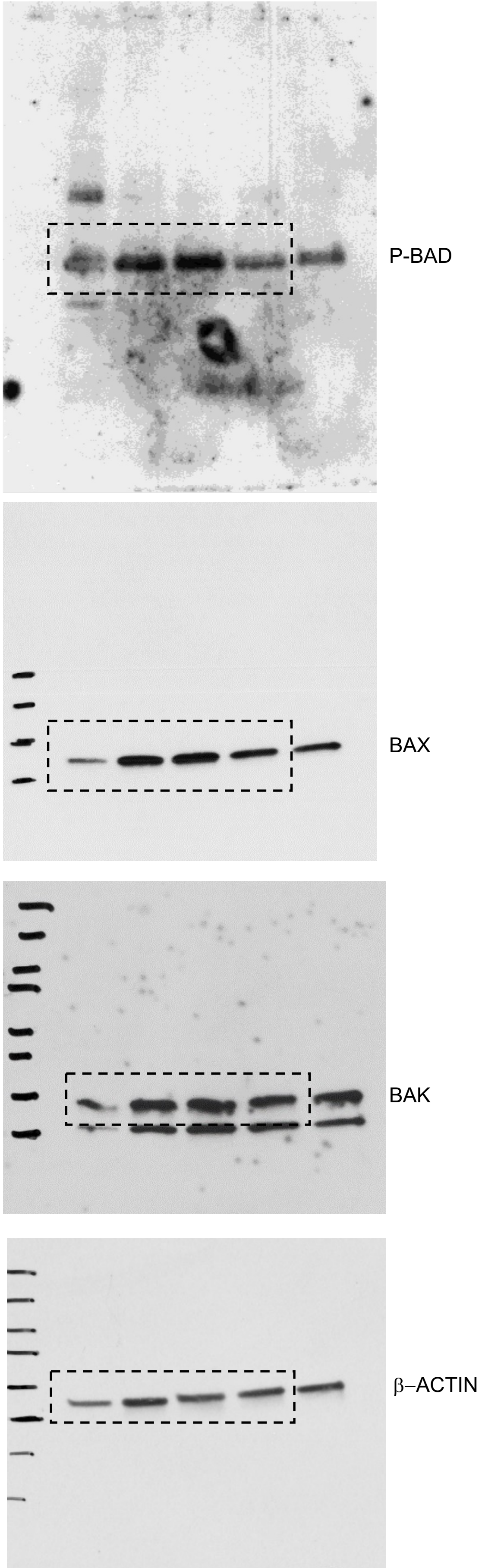

Supplementary Figure 11A

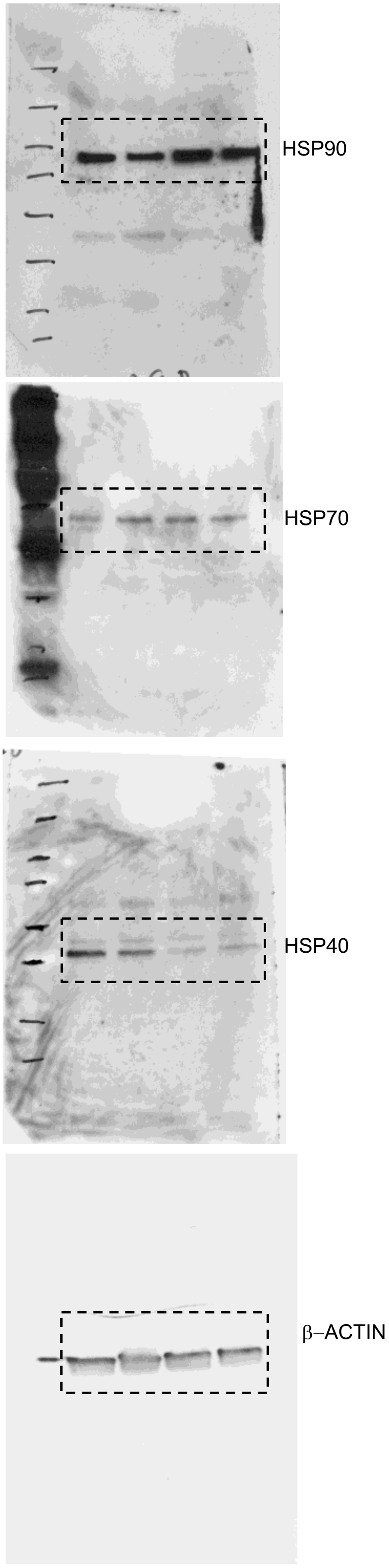

Supplement: Unedited blot and gel images [file jci-135-177342-s036.pdf]
